# Homicide Rates Across County, Race, Ethnicity, Age, and Sex in the US: A Global Burden of Disease Study

**DOI:** 10.1001/jamanetworkopen.2024.62069

**Published:** 2025-02-27

**Authors:** GBD US Health Disparities Collaborators, Paula D. Strassle, Parkes Kendrick, Mathew M. Baumann, Yekaterina O. Kelly, Zhuochen Li, Chris Schmidt, Dillon O. Sylte, Kelly Compton, Gregory J. Bertolacci, Wichada La Motte-Kerr, Farah Daoud, Mohsen Naghavi, Erik J. Rodriquez, George A. Mensah, Christopher J. L. Murray, Ali H. Mokdad, Laura Dwyer-Lindgren, Eliseo J. Pérez-Stable

**Affiliations:** 1 Division of Intramural Research, National Institute on Minority Health and Health Disparities, National Institutes of Health, Bethesda, Maryland; 2 Department of Epidemiology and Biostatistics, University of Maryland, College Park; 3 Institute for Health Metrics and Evaluation, University of Washington, Seattle; 4 Department of Health Metrics Sciences, University of Washington, Seattle; 5 Epidemiology and Community Health Branch, Division of Intramural Research, National Heart, Lung, and Blood Institute, National Institutes of Health, Bethesda, Maryland; 6 Center for Translation Research and Implementation Science, National Heart, Lung, and Blood Institute, National Institutes of Health, Bethesda, Maryland; 7 Office of Director, National Institute on Minority Health and Health Disparities, National Institutes of Health, Bethesda, Maryland

## Abstract

**Importance:**

Homicide is one of the leading causes of death in the US, especially among adolescents and adults younger than 45 years. While geographic, racial and ethnic, and sex differences in homicide rates have been documented, a comprehensive assessment across all sociodemographics is needed.

**Objective:**

To assess variation in US homicide rates from 2000 to 2019 across geographic location, race and ethnicity, sex, and age.

**Design, Setting, and Participants:**

This cross-sectional study used deidentified death records from the National Vital Statistics System and population estimates from the National Center for Health Statistics for all individuals living in the US from January 1, 2000, to December 31, 2019. Data analysis was completed in April 2023.

**Exposure:**

County, race and ethnicity (American Indian or Alaska Native, Asian or Pacific Islander, Black, Hispanic or Latino, and White), age (0-14, 15-24, 25-44, 45-64, and ≥65 years), and sex (female and male) as reported on death certificates.

**Main Outcomes and Measures:**

The main outcome was homicide rates per 100 000 individuals. Validated small-area estimation models were used to estimate county-level homicide rates by race and ethnicity, age, and sex (50 unique populations). Estimates were corrected for race and ethnicity misclassification on death certificates and were age standardized.

**Results:**

Between 2000 and 2019, there were 367 827 (95% uncertainty interval [UI], 366 683-369 046) homicides in the US, with decedents most commonly being male (77.7% [95% UI, 77.5%-77.8%]), aged 15 to 44 years (69.8% [95% UI, 69.6%-69.9%]), and Black (46.0% [95% UI, 45.5%-46.5%]). The highest homicide rates were among Black males aged 15 to 24 years (74.6 [95% UI, 72.3-77.0] per 100 000 population) and 25 to 44 years (70.0 [95% UI, 68.4-71.4] per 100 000 population) followed by American Indian and Alaska Native males aged 15 to 24 years (24.5 [95% UI, 19.2-31.0] per 100 000 population) and 25 to 44 years (33.5 [95% UI, 28.6-38.8] per 100 000 population). Homicide rates higher than 100 deaths per 100 000 population among American Indian or Alaska Native or Black males aged 15 to 44 years were observed in 143 counties; more than 25% of counties with this homicide level among Black males were in Arkansas, Louisiana, and Mississippi, and all counties with this homicide level among American Indian or Alaska Native males were in North Carolina. Despite national homicide rates remaining stable over the study period (6.1 [95% UI, 6.0-6.2] per 100 000 population for both years; incidence rate difference, 0.04 [95% UI, −0.16 to 0.07]), homicide rates increased in most counties (range, 1631 of 3051 [53.5%] to 1406 of 1488 [94.5%]) among American Indian or Alaska Native, Black, and White males and females younger than 65 years.

**Conclusions and Relevance:**

In this cross-sectional study of US homicide rates, substantial variation was found across and within county, race and ethnicity, sex, and age groups; American Indian and Alaska Native and Black males aged 15 to 44 years had the highest rates of homicide. The findings highlight several populations and places where homicide rates were high, but awareness and violence prevention remains limited.

## Key Points

**Question:**

Did homicide rates vary across county, race, ethnicity, age, and sex in the US from 2000 to 2019?

**Findings:**

In this cross-sectional study of 367 827 homicides, the largest county-level variation in homicide rates was observed among American Indian or Alaska Native and Black males aged 25 to 44 years. Homicide rates increased in most counties for American Indian or Alaska Native, Black, and White females and males younger than 65 years despite national trends being stable.

**Meaning:**

These findings suggest that the mechanisms associated with risk for violence are multifaceted.

## Teaser

This cross-sectional study examines homicide rates and trends in the US from 2000 to 2019 by county, race and ethnicity, and sex.

## Introduction

Homicide is one of the leading causes of death in the US.^1^ Racial and ethnic disparities in homicide are well documented^1^; however, research has largely focused on Black, Latino, and White populations. The underrepresentation of American Indian or Alaska Native individuals is particularly problematic, as national homicide rates in this group have been increasing.^2^ Differences in homicide rates across geographic location^3,4^ and sex^5^ have also been observed, but to our knowledge, there have been no comprehensive assessments looking at the intersection of geographic location, race and ethnicity, sex, and age that could be used to identify high-risk populations and places for targeted intervention.

Firearms are used in more than 75% of homicides in the US,^3^ but research has also been largely limited to Black, Latino, and White populations. Many studies have combined all firearm-related deaths (homicide, suicide, and unintentional), which masks differences across geographic location, race and ethnicity, and sex since risk varies substantially across these mechanisms. Assessing trends in firearm-related homicide specifically and across multiple sociodemographics is needed to reduce firearm violence and mortality. Thus, the purpose of this study was to estimate county-level homicide rates (overall and firearm-related) in the US across race and ethnicity, sex, and age categories from 2000 to 2019 and identify variation in rates and trends over time both between and within populations.

## Methods

In this cross-sectional study using deidentified death records from the US National Vital Statistics System and population estimates from the US National Center for Health Statistics (NCHS) from January 1, 2000, to December 31, 2019, we concurrently analyzed all causes of death by county, race and ethnicity, sex, age, and year. This study was approved by the University of Washington and followed the Strengthening the Reporting of Observational Studies in Epidemiology (STROBE) reporting guideline. Informed consent was not required because the study used deidentified data and was retrospective.

Race and ethnicity were ascertained from death certificates and combined into a single measure with 5 mutually exclusive populations: American Indian or Alaska Native, Asian or Pacific Islander (hereafter, *Asian*), Black, Hispanic or Latino (hereafter, *Latino*), and White. For individuals with multiple racial identities, the bridged race imputed by the NCHS was used. Due to constraints in the underlying data, we combined the Asian and Native Hawaiian or Pacific Islander populations; however, we refer to this combined population as Asian since estimates for this combined population predominantly reflect the experience of the Asian population, which is almost 20 times larger than the Native Hawaiian or Pacific Islander population in the US. Because some county boundaries changed over the analysis period, we created temporally stable geographic units, which reduced the number of areas analyzed from 3143 (the number of counties or county equivalents [eg, parishes] in 2019) to 3110 counties or combined county units.

Causes of death were classified using the Global Burden of Diseases, Injuries, and Risk Factors (GBD) 2021 study hierarchy. Interpersonal violence deaths (ie, homicides) were identified using *International Statistical Classification of Diseases and Related Health Problems, 10th Revision* codes X85 to Y09.9 and Y87.1, and firearm-related homicides were identified using a subset of those codes (X93-X95.9). We applied algorithms developed for GBD to reassign garbage codes (cause-of-death codes that referred to an intermediate or immediate cause of death, were otherwise implausible, or were insufficiently specific) to the likely true underlying cause of death.^6,7^

### Statistical Analyses

First, we used small-area estimation models to estimate the underlying homicide mortality rate (overall and firearm related) by county, race and ethnicity, sex, age, and year. Data on income, population density, postsecondary educational level, poverty, and birthplace (inside vs outside the US) by county and race and ethnicity were also incorporated as covariates to better inform the estimates. Next, we used published race and ethnicity misclassification ratios to adjust estimates of the mortality rate derived from the small-area model. Third, to guarantee consistency and ensure that misclassification adjustments did not change overall mortality rates in each county, we performed post hoc calibration using a 2-stage iterative proportional fitting algorithm. Estimates were then age-standardized to the 2010 US census.

We masked (ie, “do not depict”) homicide rates for county and racial and ethnic population combinations that had a mean annual population less than 1000 because model performance declined notably below this threshold. The details of the data sources used and our modeling approach, including model validation and performance metrics, and references have been published elsewhere.^8,9^ All analyses were performed using R, version 3.6.1 (R Project for Statistical Computing). Data analysis was completed in April 2023.

## Results

### National Homicide Rates

After excluding county and racial and ethnic population combinations with a mean annual population less than 1000, we report estimates for 3079 of 3110 counties (99.0%) for the total population, 474 (15.2%) for the American Indian or Alaska Native population, 667 (21.4%) for the Asian population, 1488 (47.8%) for the Black population, 1478 (47.5%) for the Latino population, and 3051 (98.1%) for the White population. A total of 82.2% of American Indian or Alaska Native, 97.4% of Asian, 99.0% of Black, 98.8% of Latino, and >99.9% of White individuals lived in counties with unmasked estimates.

Between 2000 and 2019, there were 367 827 (95% uncertainty interval [UI], 366 683-369 046) homicides in the US. Decedents were most likely to be male (77.7% [95% UI, 77.5%-77.8%]), aged 15 to 44 years (69.8% [95% UI, 69.6%-69.9%]), and Black (46.0% [95% UI, 45.5%-46.5%]).

**Figure 1.**
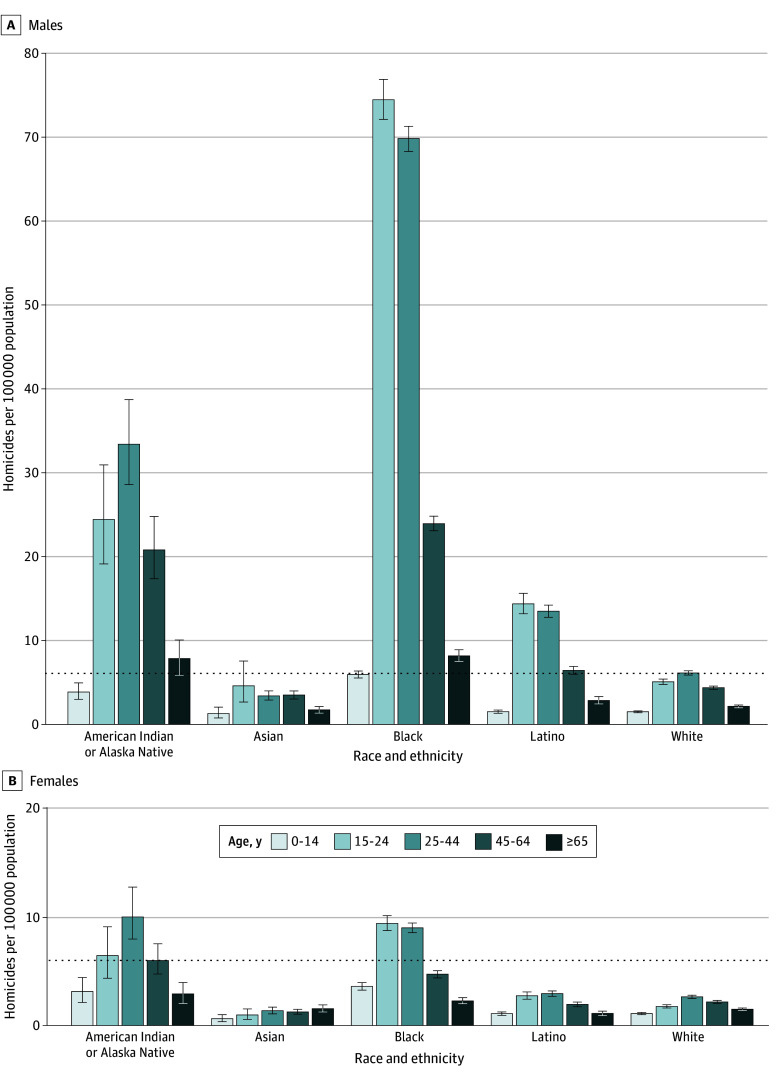
National Homicide Rates in 2019 by Race and Ethnicity, Age, and Sex Error bars represent the 95% uncertainty intervals and dashed horizontal lines, the national average.

In 2019, the overall homicide rate was 6.1 (95% UI, 6.0-6.2) per 100 000 population; however, differences were observed across race and ethnicity, sex, and age (Figure 1). The highest homicide rates, by a substantial margin, were among Black males aged 15 to 24 years (74.6 [95% UI, 72.3-77.0] per 100 000 population) and 25 to 44 years (70.0 [95% UI, 68.4-71.4] per 100 000 population) followed by American Indian and Alaska Native males aged 15 to 24 years (24.5 [95% UI, 19.2-31.0] per 100 000 population) and 25 to 44 years (33.5 [95% UI, 28.6-38.8] per 100 000 population). Homicide rates in females were highest among American Indian or Alaska Native females aged 25 to 44 years (10.2 [95% UI, 8.1-13.0] per 100 000 population), Black females aged 15 to 24 years (9.6 [95% UI, 8.9-10.3] per 100 000 population), and Black females aged 25 to 44 years (9.1 [95% UI, 8.7-9.6] per 100 000 population). Despite males generally having higher homicide rates, American Indian or Alaska Native females aged 25 to 44 years and Black females aged 15 to 24 and 25 to 44 years had the 9th, 10th, and 11th highest homicide rates in the US among the 50 combinations of race and ethnicity, sex, and age considered in this study; these rates were higher than those among all Asian and White males.

**Figure 2.**
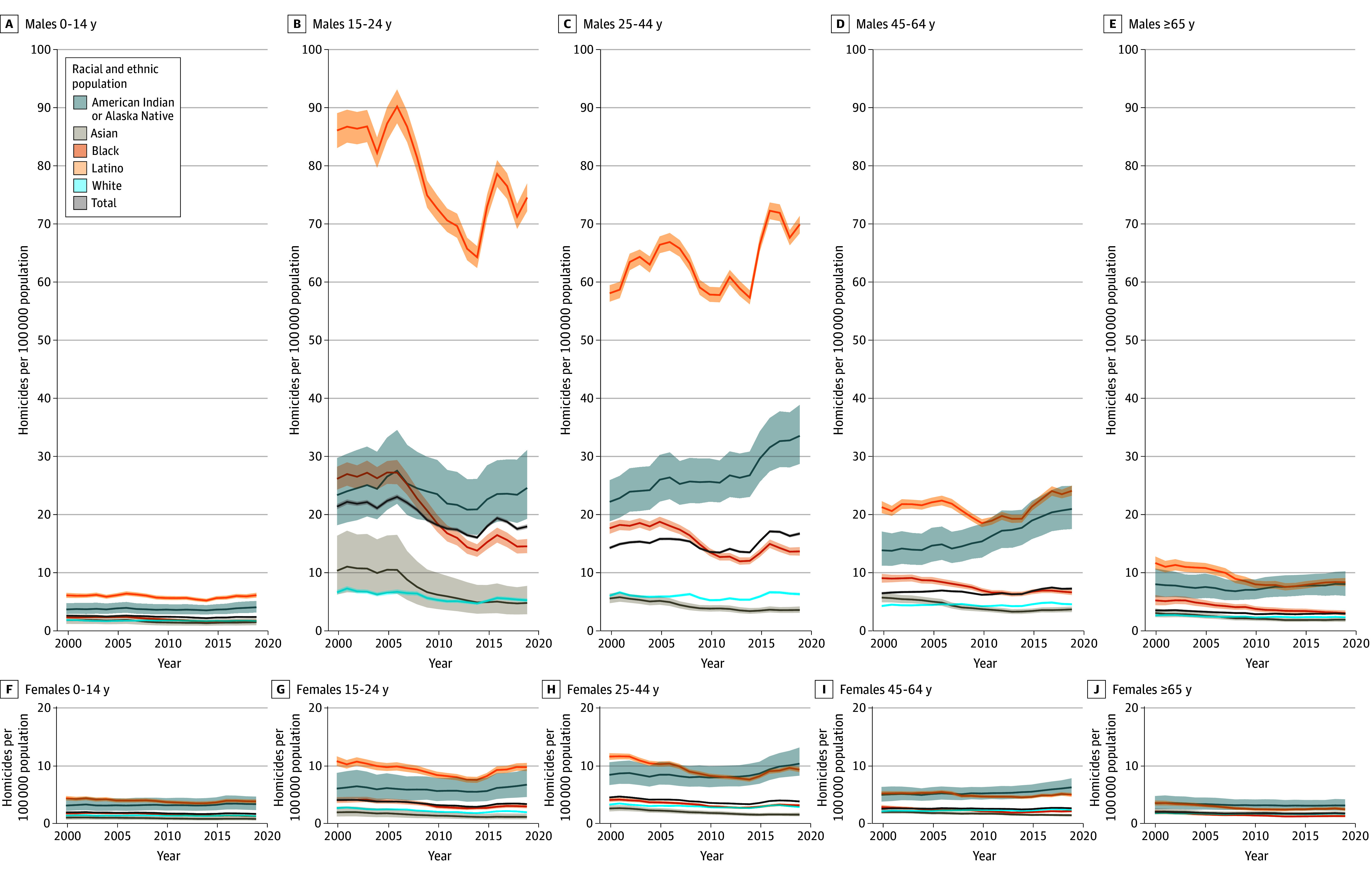
Changes in National Homicide Rates From 2000 to 2019 by Race and Ethnicity, Age, and Sex Shaded areas represent the 95% uncertainty intervals.

Between 2000 and 2019, the national homicide rate did not change (6.1 [95% UI, 6.0-6.2] per 100 000 population for both years; incidence rate difference [IRD], −0.04 [95% UI, −0.16 to 0.07]), but changes were found across race and ethnicity, sex, and age. Among Black males aged 15 to 24 years, homicide rates increased slightly between 2000 and 2005 before decreasing from 90.3 (95% UI, 87.4-93.3) per 100 000 population in 2006 to 64.3 (95% UI, 62.4-66.2) per 100 000 population in 2014, a 28.8% (95% UI, 26.9%-30.8%) decrease. However, in 2015, rates began increasing again, peaking at 78.7 (95% UI, 76.4-81.0) per 100 000 population in 2016 (Figure 2). Similar trends were observed among Black males aged 25 to 44 years and 45 to 64 years and American Indian or Alaska Native males aged 15 to 24 years. From 2000 to 2019, homicide rates steadily increased among American Indian or Alaska Native males aged 25 to 44 years (from 22.1 [95% UI, 18.7-25.8] to 33.5 [95% UI, 28.6-38.80] per 100 000 population) and 45 to 64 years (from 13.7 [95% UI, 11.1-16.9] to 20.8 [95% UI, 17.4-24.8] per 100 000 population). Homicide rates increased slightly among American Indian or Alaska Native females younger than 65 years (IRDs ranged from 0.24 [95% UI, −0.54 to 1.05] to 1.93 [95% UI, 0.10-3.79]) and White females aged 45 to 64 years (IRD, 0.25 [95% UI, 0.07-0.44]) but decreased for all other female populations (IRDs ranged from −2.26 [95% UI, −2.99 to −1.56] to −0.11 [95% UI, −0.24 to 0.03]).

### County-Level Homicide Rates in 2019

Substantial variation in county-level homicide rates was observed across almost all racial and ethnic, sex, and age populations (eFigure 1 in Supplement 1). In 2019, among males and overall, the largest county-level variation was seen among Black males aged 25 to 44 years (2.8 [95% UI, 0.9-6.0] to 348.9 [95% UI, 313.2-385.5] per 100 000 population) followed by Black males aged 15 to 24 years (4.5 [95% UI, 1.6-9.0] to 235.0 [95% UI, 212.3-259.0] per 100 000 population) and American Indian or Alaska Native males aged 25 to 44 years (3.7 [95% UI, 1.3-8.9] to 129.9 [95% UI, 103.3-156.3] per 100 000 population) and 15 to 24 years (2.9 [95% UI, 0.7-7.6] to 101.9 [95% UI, 74.2-131.7] per 100 000 population). Among females, the largest variation was observed among American Indian or Alaska Native females aged 25 to 44 years (1.5 [95% UI, 0.4-3.8] to 58.0 [95% UI, 27.7-99.9] per 100 000 population) followed by Black females aged 25 to 44 years (1.6 [95% UI, 0.6-3.5] to 39.2 [95% UI, 30.7-49.1] per 100 000 population) and American Indian or Alaska Native females aged 45 to 64 years (0.9 [95% UI, 0.2-2.6] to 33.1 [95% UI, 11.7-63.1] per 100 000 population).

**Figure 3.**
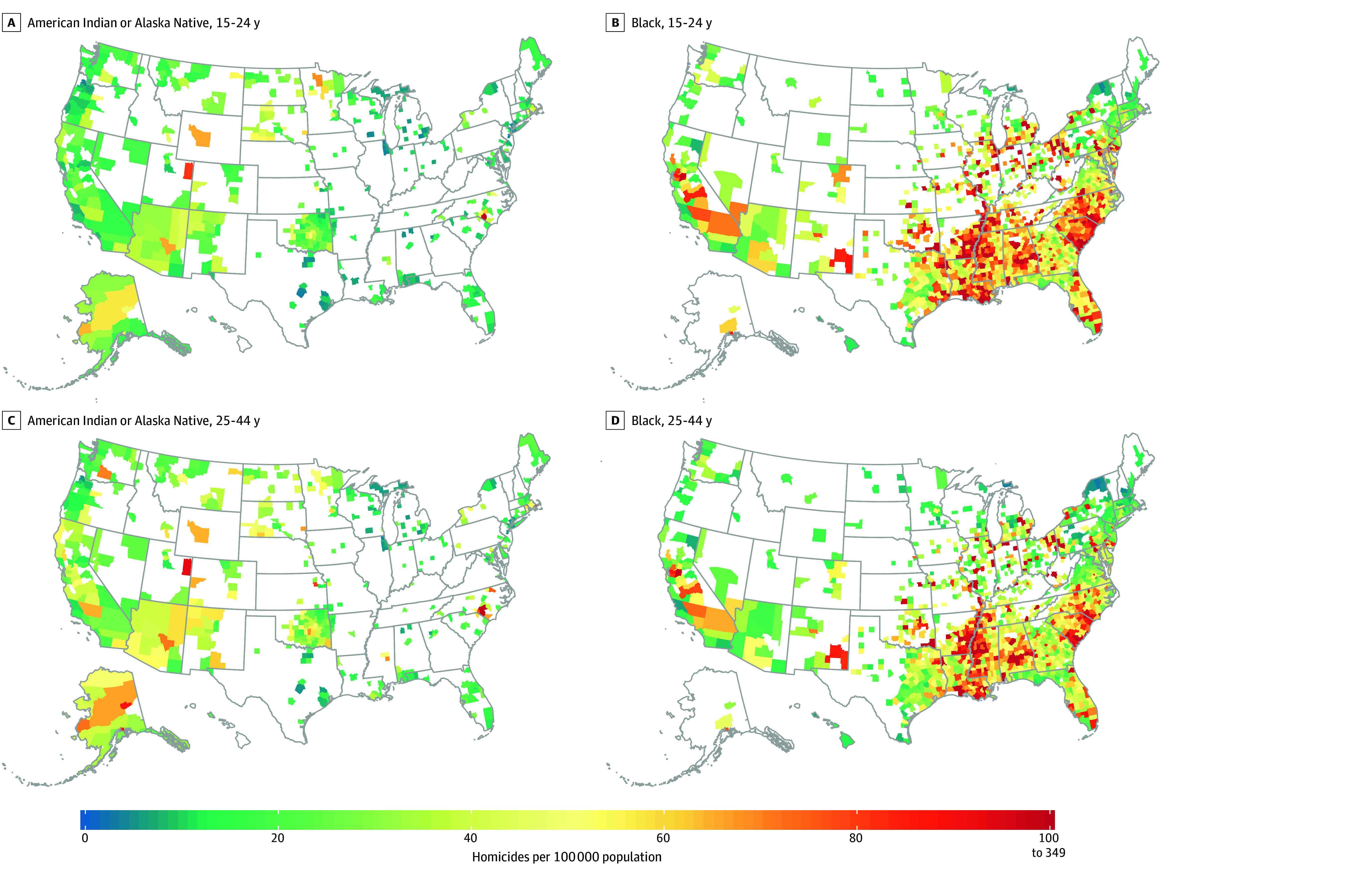
Age-Standardized, County-Level Homicide Rates in 2019 Among American Indian or Alaska Native and Black Males Aged 15 to 24 or 25 to 44 Years Estimates were masked (white) for counties with a mean annual population of less than 1000 because model performance declined notably below this threshold.

Homicide rates higher than 100 per 100 000 population among American Indian or Alaska Native or Black males aged 15 to 44 years were observed in 143 unique counties: 118 counties for Black males aged 15 to 24 years, 90 counties for Black males aged 25 to 44 to years, 3 counties for American Indian or Alaska Native males aged 25 to 44 years, and 1 county for both American Indian or Alaska Native males aged 15 to 24 years and Black males aged 45 to 64 years; a full list can be found in eTable 1 in Supplement 1. Over one-quarter of counties with homicide rates higher than 100 per 100 000 population for Black males aged 15 to 24 years (30 of 118 counties [25.4%]) or 25 to 44 years (27 of 90 counties [30.0%]) were in Arkansas, Louisiana, or Mississippi (Figure 3). For American Indian or Alaska Native males aged 15 to 24 or 25 to 44 years, the counties with homicide rates higher than 100 per 100 000 population were all located in North Carolina (Figure 3). Maps for the other race and ethnicity, sex, and age populations can be found in eFigures 2 and 3 in Supplement 1.

Among females, the 4 highest homicide rates were in American Indian or Alaska Native females aged 25 to 44 years living in Fairbanks North Star Borough, Alaska (58.0 [95% UI, 27.7-99.9] per 100 000 population); Matanuska-Susitna Borough, Alaska (47.0 [95% UI, 24.0-79.0] per 100 000 population); Anchorage Municipality, Alaska (41.6 [95% UI, 22.5-65.9] per 100 000 population); and a combined county unit that included multiple boroughs in the southernmost point of Alaska (39.8 [95% UI, 16.7-72.6] per 100 000 population). Alaskan counties also had the highest homicide rates for American Indian or Alaska Native females aged 15 to 24, 45 to 64, or 65 or more years as well as Asian and Latina females aged 15 to 44 years. County-level homicide rates were also high in New Mexico and Colorado for Latina females aged 15 to 24 and 25 to 44 years.

The fifth highest county-level rate was among Black females aged 25 to 44 years in Saint Louis, Missouri (39.2 [95% UI, 30.7-49.1] per 100 000 population). Overall, counties with high homicide rates among Black females were largely located along the Mississippi River (Arkansas, Missouri, and Tennessee) and in Ohio.

### Changes in County-Level Homicide Rates

**Figure 4.**
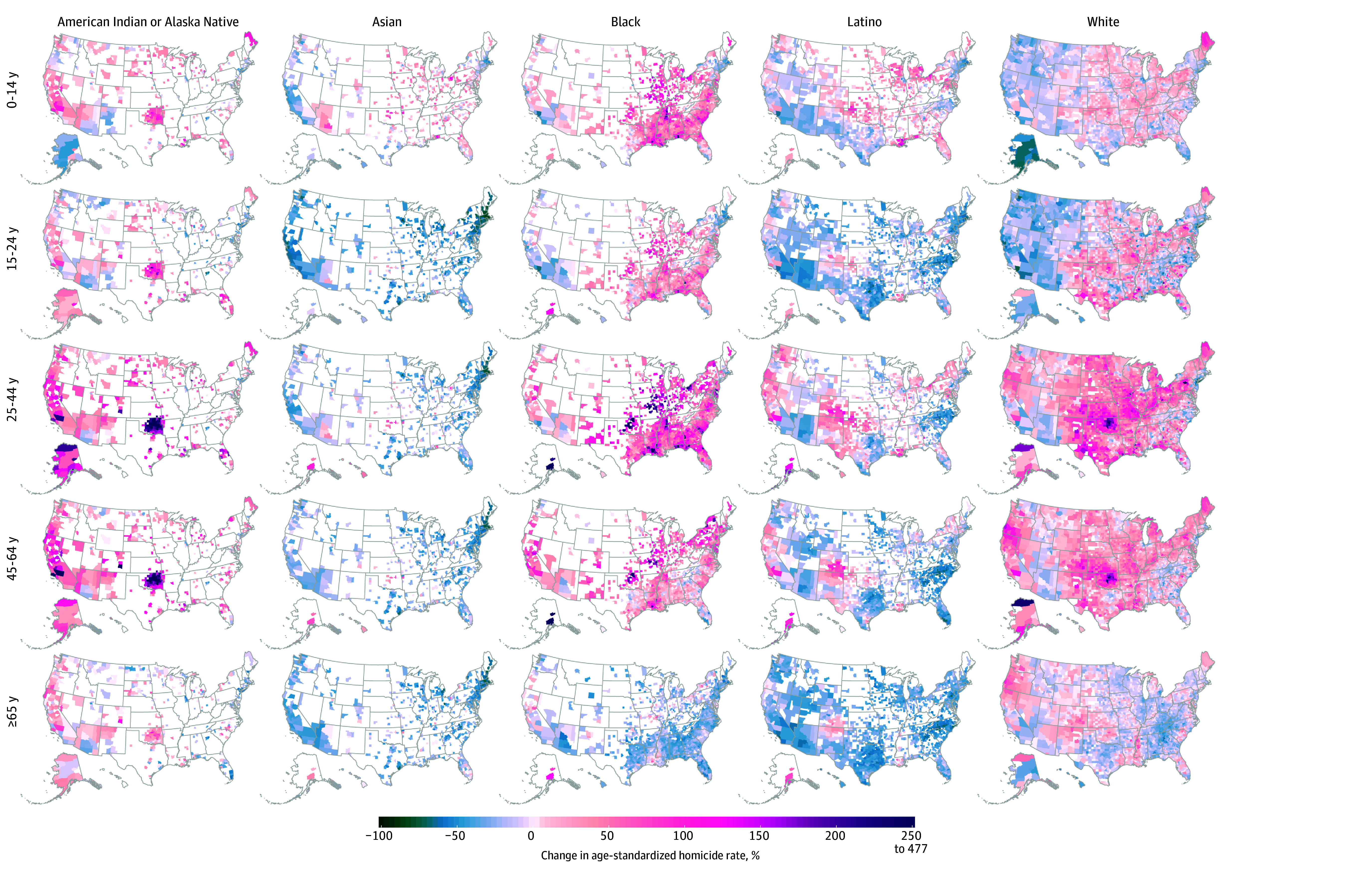
Changes in County-Level Homicide Rates Between 2000 and 2019 Among Males by Race and Ethnicity and Age Estimates were masked (white) for counties with a mean annual population of less than 1000 because model performance declined notably below this threshold.

Homicide rates among American Indian or Alaska Native, Black, and White males increased in a majority of counties across all age groups (1631 of 3051 [53.5%] to 1406 of 1488 [94.5%]) except among Black males and White males aged 65 years or older, among whom 151 of 1488 counties (10.1%) and 943 of 3051 counties (30.9%), respectively, saw an increase in homicide (Figure 4 and eTable 2 in Supplement 1). Conversely, among Asian males aged 15 years or older and Latino males aged 15 to 24 years or 45 years or older, most counties saw decreases in homicide rates during the 2000-2019 study period (1187 of 1478 [80.3%] to 650 of 667 [97.5%]). A smaller majority of counties also saw homicide rates decrease among Latino males aged 25 to 44 years (788 of 1478 [53.3%]).

Among females, homicide rates increased in the majority of counties among American Indian or Alaska Native females aged 15 to 24 years (273 of 474 [57.6%]), 25 to 44 years (360 of 474 [75.9%]), and 45 to 64 years (371 of 474 [78.3%]); Black females aged 15 to 24 years (983 of 1488 [66.1%]) and 45 to 64 years (953 of 1488 [64.0%]); and White females 45 to 64 years (2619 of 3051 [85.8%]) (eFigure 4 and eTable 2 in Supplement 1). Homicide rates decreased in most counties across all ages among Asian females (597 [89.5%] to 659 [98.8%] of 667 counties) and Latina females (1095 [74.1%] to 1470 [99.5%] of 1478 counties).

### Firearm-Related Homicide Rates

Overall, 76.1% (95% UI, 75.5%-76.6%) of all homicides were firearm related in 2019; however, across race and ethnicity, age, and sex categories, that proportion ranged from 22.5% (95% UI, 19.5%-26.1%) to 96.0% (95% UI, 95.5%-96.4%) (eFigure 5 in Supplement 1). The highest proportions were seen among Black males aged 15 to 24 years (96.0% [95% UI, 95.5%-96.4%]) and 25 to 44 years (90.9% [95% UI, 90.3%-91.5%]), Latino males aged 15 to 24 years (89.3% [95% UI, 88.1%-90.5%]), Black females aged 15 to 24 years (84.3% [95% UI, 82.7%-86.0%]), and White males aged 15 to 24 years (83.7% [95% UI, 82.1%-85.2%]). The lowest proportions were observed among females and males aged 0 to 14 years (range, 22.5% [95% UI, 9.5%-26.1%] to 42.4% [95% UI, 40.1%-44.6%]). Given the high proportion of homicides that were firearm related, firearm-related homicide rates (overall and county level) were similar to overall homicide estimates (eFigures 6-8 in Supplement 1).

Nationally, firearm-related homicide rates increased modestly between 2000 and 2019 (IRD, 0.51 [95% UI, 0.42-0.59]). Similar national trends were also observed for each race and ethnicity, sex, and age population (eFigure 9 in Supplement 1). More counties saw increases or more substantial increases in firearm-related homicides compared with overall homicides across all 50 populations (eFigures 10 and 11 in Supplement 1).

## Discussion

Homicide and violence (overall and firearm related) are a public health crisis in the US. To our knowledge, this analysis represents the most comprehensive assessment of US homicide rates (overall and firearm related) to date and estimates county-specific rates across 50 unique race and ethnicity, sex, and age populations in the US. We found that racial and ethnic disparities in homicide were prevalent and substantial across the country—both in urban and nonurban areas and particularly among American Indian or Alaska Native and Black females and males. Studies have shown that neighborhood factors, like structural racism,^10,11,12,13^ social structure,^14,15^ poverty,^16,17,18^ lack of opportunity and perceived hopelessness,^19^ and other social determinants of health,^20,21^ which disproportionately impact marginalized communities, are associated with higher risk of homicide. Homicide also has substantial impacts on family members, friends, and communities and may contribute to health disparities. Exposure to homicide has been associated with worse mental health,^22,23^ and neighborhoods with higher rates of homicide and gun violence have poorer collective health, including poorer health behaviors, worse mental health, and higher risk of chronic conditions and disability.^24^ The relationship between neighborhood violence and health may also be reciprocal, with poorer community health being a risk factor for increased rates of violence.^24^

Even within racial and ethnic, sex, and age groups, we found considerable geographic variation in homicide rates across the US. Some of these differences are likely explained by geographic differences in social determinants of health. While many consider homicide and gun violence to be primarily urban problems, our study and others^25,26^ have found that homicide rates vary substantially across US cities and that some cities have lower homicide rates than the national average.^26^ In our analysis, some of the highest homicide rates were in nonmetropolitan counties. To date, there has been little focus on addressing homicide among rural communities. For American Indian or Alaska Native males, high rates also appeared to concentrate in counties that included Indian reservations. Indian reservations have the highest poverty rates in the US, with unemployment rates up to 50% and roughly 1 in 4 American Indian or Alaska Native individuals on reservations living in poverty.^27^ Improving economic opportunities on reservations, which potentially could reduce homicide rates, is cumbersome and requires navigating complex federal policies that date back to the dispossession of Native communities and the creation of reservations in the mid-1800s.^27^

It is estimated that 40% to 50% of female homicides in the US can be attributed to intimate partner violence (IPV).^28,29^ IPV and IPV-related homicide are more likely to impact younger females and racial and ethnic minority populations.^30^ High rates of IPV may explain why the highest homicide rates among American Indian or Alaska Native, Asian, and Latina females were found in Alaskan counties and why the highest homicide rates among females were observed among American Indian or Alaska Native females aged 25 to 44 years in Alaska. Almost half (48%) of adult females living in Alaska have reported experiencing IPV during their lifetime.^31^ Federal and state laws in place to prevent individuals with domestic abuse charges from acquiring firearms have been shown to reduce homicide rates among females; however, the effectiveness of these laws has been limited due to implementation issues.^30,32^ Two major concerns include the following: (1) fewer than half of males who kill their partners have been arrested for domestic violence prior to the homicide (and therefore are not impacted by the law), and (2) there are several characteristics of the federal background check system that limit the ability to identify individuals with domestic violence charges.^33^ At least 1 study also found that these laws only reduced intimate partner homicide rates among White females.^34^ In addition, while the Centers for Disease Control and Prevention has developed best practice guidelines to help prevent IPV,^35^ recommendations based on existing evidence largely focus on behavioral interventions for IPV perpetrators and victims rather than the structural factors associated with higher risk of committing or experiencing IPV.

For most populations in the US, the majority of homicides were found to be firearm related, which explains why a substantial focus of violence prevention has been on gun control laws. Both gun ownership and firearm-related deaths (which include homicide, suicide, and accidental deaths) are higher in the US compared with other high-income countries,^36^ and gun ownership has been consistently associated with increased homicide risk.^37^ In both the US^38^ and internationally,^39^ gun control laws have been associated with reductions in homicide. However, gun control laws can target sales and purchasing requirements, restrictions on gun ownership, or gun storage requirements, and the heterogeneity makes it difficult to identify effective strategies.^39,40^ Additionally, the effectiveness of gun control laws implemented by individual cities and states is also impacted by gun control laws (or lack thereof) in neighboring communities; at least 1 study found that 65% of recovered firearms in the most restrictive gun control states originated from other states—higher proportion than observed in less restrictive states (44%).^41^ These nuances are rarely acknowledged when discussing the effectiveness of gun control in the US.

Research on non–gun control violence prevention remains somewhat limited. Gun buy-back programs have been found to have minimal impact on homicide rates, especially when implemented in nonisolated communities.^42^ Community-based violence prevention programs (eg, violence interrupters) can be effective when they are culturally appropriate, address social determinants of health, and are adequately funded,^42^ but overall, these programs have had mixed results.^42,43,44^ Increased spending in public health and social services has been correlated with lower homicide rates in the US,^45^ and randomized trials have found that remediating vacant lots^46^ and installing street lighting^47^ are effective in reducing homicide rates in cities. The number of off-premises alcohol outlets and pawn firearm dealers has also been linked to higher rates of violence and homicide,^48^ suggesting that changes to zoning policy could also reduce violence. Investments to improve and maintain neighborhoods and increase access to affordable housing, green space, employment opportunities, education, and health care may be the best long-term deterrents for violence in the US but require leveraging leadership to generate support and allocate appropriate resources to largely underfunded communities.

### Limitations

This analysis has a few limitations that have been discussed elsewhere, including potential errors in the underlying data, the degree of model smoothing, and racial and ethnic misclassification corrections.^8,9^ Additionally, while we developed algorithms to address insufficiently specific or implausible underlying cause of death codes, some misclassification may have still existed. There may also have been some bias in the reported race and ethnicity, as individuals who die by homicide are more likely to be classified as Black by those filling in death certificates.^49^ We were also limited in our ability to look at homicide rates across race and ethnicity.

## Conclusions

This cross-sectional study found that homicide has remained a substantial public health crisis in the US, especially among American Indian or Alaska Native and Black females and males. Disparities and geographic variation in homicide rates across race and ethnicity, sex, and age found in this study suggest that the mechanisms associated with risk for violence are multifaceted. These findings also highlight several populations (eg, American Indian or Alaska Native females) and places (rural communities) with high homicide rates, but awareness and violence prevention strategies remain limited. Both community-based interventions and policy to address systemic factors must be implemented to target specific populations in these areas with high risk to have meaningful effects.

## Notes

Supplement 1. **eFigure 1.** Age-Standardized, County-Level Homicide Rates in 2019 by Race and Ethnicity, Sex, and Age **eFigure 2.** Map of Age-Standardized, County-Level Homicide Rates in 2019 by Race and Ethnicity and Age, Males **eFigure 3.** Map of Age-Standardized, County-Level Homicide Rates in 2019 by Race and Ethnicity and Age, Females **eFigure 4.** Map of Age-Standardized, County-Level Changes in Homicide Rates From 2000 to 2019 by Race and Ethnicity and Age, Females **eFigure 5.** Percentage of all Homicides That Were Firearm-Related in 2019, by Race and Ethnicity, Sex, and Age **eFigure 6**. Age-Standardized, National Firearm-Related Homicide Rates in 2019, by Race and Ethnicity, Sex, and Age **eFigure 7**. Map of Age-Standardized, County-Level Firearm-Related Homicide Rates in 2019 by Race and Ethnicity and Age, Males **eFigure 8**. Map of Age-Standardized, County-Level Firearm-Related Homicide Rates in 2019 by Race and Ethnicity and Age, Females **eFigure 9**. Changes in Age-Standardized, National Firearm-Related Homicide Rates From 2000 to 2019, by Race and Ethnicity, Age, and Sex **eFigure 10**. Changes in Age-Standardized, County-Level, Firearm-Related Homicide Rates Between 2000 and 2019 Among Males, by Race and Ethnicity and Age **eFigure 11**. Changes in Age-Standardized, County-Level, Firearm-Related Homicide Rates Between 2000 and 2019 Among Females, by Race and Ethnicity and Age **eTable 1.** Counties With Age-Standardized, Homicide Rates Greater than 100 Per 100 000 Population in 2019 Among American Indian or Alaska Native Males Aged 15 to 44 Years and Black Males Aged 15 to 64 Years **eTable 2.** Median Changes in Age-Standardized, County-Level Homicide Rates and the Proportion of Counties That Saw Increases and Decreases Between 2000 and 2019, by Race and Ethnicity, Sex, and Age

Supplement 2.
Data Sharing Statement

## References

[1] Wilson RF, Liu G, Lyons BH, . Surveillance for violent deaths—National Violent Death Reporting System, 42 states, the District of Columbia, and Puerto Rico, 2019. MMWR Surveill Summ. 2022;71(6):1-40. doi:10.15585/mmwr.ss7106a1 35588398 PMC9129903

[2] Kegler SR, Dahlberg LL, Vivolo-Kantor AM. A descriptive exploration of the geographic and sociodemographic concentration of firearm homicide in the United States, 2004-2018. Prev Med. 2021;153:106767. doi:10.1016/j.ypmed.2021.106767 34416223 PMC8667260

[3] Smart R, Schell TL, Morral AR, Nicosia N. Geographic disparities in rising rates of firearm-related homicide. N Engl J Med. 2022;387(2):189-191. doi:10.1056/NEJMc2203322 35830647 PMC9746702

[4] Baller RD, Anselin L, Messner SF, Deane G, Hawkins DF. Structural covariates of US county homicide rates: Incorporating spatial effects. Criminology. 2001;39(3):561-588. doi:10.1111/j.1745-9125.2001.tb00933.x

[5] Fridel EE, Fox JA. Gender differences in patterns and trends in US homicide, 1976–2017. Violence Gend. 2019;6(1):27-36. doi:10.1089/vio.2019.0005

[6] Naghavi M, Makela S, Foreman K, O’Brien J, Pourmalek F, Lozano R. Algorithms for enhancing public health utility of national causes-of-death data. Popul Health Metr. 2010;8:9. doi:10.1186/1478-7954-8-9 20459720 PMC2873308

[7] GBD 2021 Causes of Death Collaborators. Global burden of 288 causes of death and life expectancy decomposition in 204 countries and territories and 811 subnational locations, 1990-2021: a systematic analysis for the Global Burden of Disease Study 2021. Lancet. 2024;403(10440):2100-2132. doi:10.1016/S0140-6736(24)00367-2 38582094 PMC11126520

[8] GBD US Health Disparities Collaborators. Life expectancy by county, race, and ethnicity in the USA, 2000-19: a systematic analysis of health disparities. Lancet. 2022;400(10345):25-38. doi:10.1016/S0140-6736(22)00876-5 35717994 PMC9256789

[9] GBD US Health Disparities Collaborators. Cause-specific mortality by county, race, and ethnicity in the USA, 2000-19: a systematic analysis of health disparities. Lancet. 2023;402(10407):1065-1082. doi:10.1016/S0140-6736(23)01088-7 37544309 PMC10528747

[10] Conrick KM, Adhia A, Ellyson A, . Race, structural racism and racial disparities in firearm homicide victimisation. Inj Prev. 2023;29(4):290-295. doi:10.1136/ip-2022-044788 36564165

[11] Unnever JD, Stults BJ, Messner SF. Structural racism and criminal violence: an analysis of state-level variation in homicide. Race Justice. 2023;13(4):433-462. doi:10.1177/21533687211015287

[12] Siegel M, Rieders M, Rieders H, . Measuring structural racism and its association with racial disparities in firearm homicide. J Racial Ethn Health Disparities. 2023;10(6):3115-3130. doi:10.1007/s40615-022-01485-2 36508134 PMC9744051

[13] Krivo LJ, Peterson RD, Kuhl DC. Segregation, racial structure, and neighborhood violent crime. AJS. 2009;114(6):1765-1802. doi:10.1086/597285 19852253

[14] Thomas SA, Harris CT, Drawve G. Exploring the influence of elements of the social and physical environment on neighborhood gun crime. Am J Crim Justice. 2022;47(3):370-398. doi:10.1007/s12103-020-09599-1

[15] Lu Y, Luo L, Santos MR. Social change and race-specific homicide trajectories: an age-period-cohort analysis. J Res Crime Delinq. 2024;61(2):224-267. doi:10.1177/00224278221129886 38344105 PMC10857748

[16] Barrett JT, Lee LK, Monuteaux MC, Farrell CA, Hoffmann JA, Fleegler EW. Association of county-level poverty and inequities with firearm-related mortality in US youth. JAMA Pediatr. 2022;176(2):e214822. doi:10.1001/jamapediatrics.2021.4822 34807238 PMC8609463

[17] Baumer EP, Fowler C, Messner SF, Rosenfeld R. Change in the spatial clustering of poor neighborhoods within US counties and its impact on homicide: an analysis of metropolitan counties, 1980-2010. Sociol Q. 2022;63(3):401-425. doi:10.1080/00380253.2020.1867485

[18] McCool WC, Codding BF. US homicide rates increase when resources are scarce and unequally distributed. Evol Hum Sci. 2023;6:e3. doi:10.1017/ehs.2023.31 38516371 PMC10955375

[19] DuRant RH, Cadenhead C, Pendergrast RA, Slavens G, Linder CW. Factors associated with the use of violence among urban Black adolescents. Am J Public Health. 1994;84(4):612-617. doi:10.2105/AJPH.84.4.612 8154565 PMC1614778

[20] Cheon C, Lin Y, Harding DJ, Wang W, Small DS. Neighborhood racial composition and gun homicides. JAMA Netw Open. 2020;3(11):e2027591. doi:10.1001/jamanetworkopen.2020.27591 33252687 PMC7705591

[21] Kim D. Social determinants of health in relation to firearm-related homicides in the United States: a nationwide multilevel cross-sectional study. PLoS Med. 2019;16(12):e1002978. doi:10.1371/journal.pmed.1002978 31846474 PMC6917210

[22] Semenza DC, Daruwala S, Brooks Stephens JR, Anestis MD. Gun violence exposure and suicide among Black adults. JAMA Netw Open. 2024;7(2):e2354953. doi:10.1001/jamanetworkopen.2023.54953 38319659 PMC10848043

[23] Leibbrand C, Hill H, Rowhani-Rahbar A, Rivara F. Invisible wounds: community exposure to gun homicides and adolescents’ mental health and behavioral outcomes. SSM Popul Health. 2020;12:100689. doi:10.1016/j.ssmph.2020.100689 33204810 PMC7653279

[24] Semenza DC, Stansfield R, Silver IA, Savage B. Reciprocal neighborhood dynamics in gun violence exposure, community health, and concentrated disadvantage in one hundred US cities. J Urban Health. 2023;100(6):1128-1139. doi:10.1007/s11524-023-00796-x 37843742 PMC10728405

[25] Levine RS, Schneid RP, Zoorob RJ, Hennekens CH. A tale of two cities: persistently high homicide rates in Baltimore City compared with significant declines in New York City. Am J Med. 2019;132(1):3-5. doi:10.1016/j.amjmed.2018.08.013 30148979

[26] Schober DJ, Hunt BR, Benjamins MR, . Homicide mortality inequities in the 30 biggest cities in the US. Am J Prev Med. 2021;60(3):327-334. doi:10.1016/j.amepre.2020.09.008 33221143

[27] Crepelle A. Federal policies trap tribes in poverty. *Human Rights*. 2023;48(2). Accessed September 9, 2024. https://www.americanbar.org/groups/crsj/publications/human_rights_magazine_home/wealth-disparities-in-civil-rights/federal-policies-trap-tribes-in-poverty/

[28] Petrosky E, Blair JM, Betz CJ, Fowler KA, Jack SPD, Lyons BH. Racial and ethnic differences in homicides of adult women and the role of intimate partner violence—United States, 2003-2014. MMWR Morb Mortal Wkly Rep. 2017;66(28):741-746. doi:10.15585/mmwr.mm6628a1 28727682 PMC5657947

[29] Brauer M, Roth GA, Aravkin AY, ; GBD 2021 Risk Factors Collaborators. Global burden and strength of evidence for 88 risk factors in 204 countries and 811 subnational locations, 1990-2021: a systematic analysis for the Global Burden of Disease Study 2021. Lancet. 2024;403(10440):2162-2203. doi:10.1016/S0140-6736(24)00933-4 38762324 PMC11120204

[30] Zeoli AM, Malinski R, Turchan B. Risks and targeted interventions: firearms in intimate partner violence. Epidemiol Rev. 2016;38(1):125-139. doi:10.1093/epirev/mxv007 26739680

[31] Johnson ID. 2020 Statewide Alaska Victimization Survey final report. Alaska Council on Domestic Violence and Sexual Assault. October 2021. Accessed October 3, 2024. https://scholarworks.alaska.edu/bitstream/handle/11122/12259/2021-10%20AVS%202020%20Final%20Report.pdf

[32] Goodyear A, Rodriguez M, Glik D. The role of firearms in intimate partner violence: policy and research considerations. J Public Health Policy. 2020;41(2):185-195. doi:10.1057/s41271-019-00198-x 31796866

[33] Messing JT, AbiNader M, Bent-Goodley T, Campbell J. Preventing intimate partner homicide: the long road ahead. Homicide Stud. 2022;26(1):91-105. doi:10.1177/10887679211048492

[34] Gray AC, Kafonek K, Parker KF. Firearms, policy, and intimate partner homicide: a structural and disaggregated examination of Black, Latina, and White female victimization. Criminology. 2024;62(2):276-299. doi:10.1111/1745-9125.12372

[35] Niolon PH, Kearns M, Dills J, et al. Intimate Partner Violence Resource For Action: A Compilation of the Best Available Evidence. Centers for Disease Control and Prevention; 2017. Accessed September 16, 2024. https://www.cdc.gov/violenceprevention/pdf/ipv-prevention-resource_508.pdf

[36] Naghavi M, Marczak LB, Kutz M, ; Global Burden of Disease 2016 Injury Collaborators. Global mortality from firearms, 1990-2016. JAMA. 2018;320(8):792-814. doi:10.1001/jama.2018.10060 30167700 PMC6143020

[37] Anglemyer A, Horvath T, Rutherford G. The accessibility of firearms and risk for suicide and homicide victimization among household members: a systematic review and meta-analysis. Ann Intern Med. 2014;160(2):101-110. doi:10.7326/M13-1301 24592495

[38] Lee LK, Fleegler EW, Farrell C, . Firearm laws and firearm homicides: a systematic review. JAMA Intern Med. 2017;177(1):106-119. doi:10.1001/jamainternmed.2016.7051 27842178

[39] Patel J, Leach-Kemon K, Curry G, Naghavi M, Sridhar D. Firearm injury—a preventable public health issue. Lancet Public Health. 2022;7(11):e976-e982. doi:10.1016/S2468-2667(22)00233-X 36334611 PMC9646976

[40] Santaella-Tenorio J, Cerdá M, Villaveces A, Galea S. What do we know about the association between firearm legislation and firearm-related injuries? Epidemiol Rev. 2016;38(1):140-157. doi:10.1093/epirev/mxv012 26905895 PMC6283012

[41] Olson EJ, Hoofnagle M, Kaufman EJ, Schwab CW, Reilly PM, Seamon MJ. American firearm homicides: the impact of your neighbors. J Trauma Acute Care Surg. 2019;86(5):797-802. doi:10.1097/TA.0000000000002212 30741886

[42] Bonne SL, Violano P, Duncan TK, . Prevention of firearm violence through specific types of community-based programming: an Eastern Association for the Surgery of Trauma evidence-based review. Ann Surg. 2021;274(2):298-305. doi:10.1097/SLA.0000000000004837 33914467

[43] Butts JA, Roman CG, Bostwick L, Porter JR. Cure violence: a public health model to reduce gun violence. Annu Rev Public Health. 2015;36:39-53. doi:10.1146/annurev-publhealth-031914-122509 25581151

[44] Buggs SA, Webster DW, Crifasi CK. Using synthetic control methodology to estimate effects of a *Cure Violence* intervention in Baltimore, Maryland. Inj Prev. 2022;28(1):61-67. doi:10.1136/injuryprev-2020-044056 33558396 PMC9019528

[45] Sipsma HL, Canavan ME, Rogan E, Taylor LA, Talbert-Slagle KM, Bradley EH. Spending on social and public health services and its association with homicide in the USA: an ecological study. BMJ Open. 2017;7(10):e016379. doi:10.1136/bmjopen-2017-016379 29025831 PMC5652551

[46] Moyer R, MacDonald JM, Ridgeway G, Branas CC. Effect of remediating blighted vacant land on shootings: a citywide cluster randomized trial. Am J Public Health. 2019;109(1):140-144. doi:10.2105/AJPH.2018.304752 30496003 PMC6301418

[47] Mitre-Becerril D, Tahamont S, Lerner J, Chalfin A. Can deterrence persist? long-term evidence from a randomized experiment in street lighting. Criminol Public Policy. 2022;21(4):865-891. doi:10.1111/1745-9133.12599

[48] Pear VA, Wintemute GJ, Jewell NP, Cerdá M, Ahern J. Community-level risk factors for firearm assault and homicide: the role of local firearm dealers and alcohol outlets. Epidemiology. 2023;34(6):798-806. doi:10.1097/EDE.0000000000001670 37708491 PMC10538383

[49] Noymer A, Penner AM, Saperstein A. Cause of death affects racial classification on death certificates. PLoS One. 2011;6(1):e15812. doi:10.1371/journal.pone.0015812 21298093 PMC3027630

