# Supplementary material for: Homicide Rates Across County, Race, Ethnicity, Age, and Sex in the US: A Global Burden of Disease Study
Source: JAMA Netw Open. 2025 Feb 27;8(2):e2462069. doi: 10.1001/jamanetworkopen.2024.62069 (PMC11868975; doi:10.1001/jamanetworkopen.2024.62069)
Supplement: Supplement 1. — eFigure 1. Age-Standardized, County-Level Homicide Rates in 2019 by Race and Ethnicity, Sex, and Age eFigure 2. Map of Age-Standardized, County-Level Homicide Rates in 2019 by Race and Ethnicity and Age, Males eFigure 3. Map of Age-Standardized, County-Level Homicide Rates in 2019 by Race and Ethnicity and Age, Females eFigure 4. Map of Age-Standardized, County-Level Changes in Homicide Rates From 2000 to 2019 by Race and Ethnicity and Age, Females eFigure 5. Percentage of all Homicides That Were Firearm-Related in 2019, by Race and Ethnicity, Sex, and Age eFigure 6. Age-Standardized, National Firearm-Related Homicide Rates in 2019, by Race and Ethnicity, Sex, and Age eFigure 7. Map of Age-Standardized, County-Level Firearm-Related Homicide Rates in 2019 by Race and Ethnicity and Age, Males eFigure 8. Map of Age-Standardized, County-Level Firearm-Related Homicide Rates in 2019 by Race and Ethnicity and Age, Females eFigure 9. Changes in Age-Standardized, National Firearm-Related Homicide Rates From 2000 to 2019, by Race and Ethnicity, Age, and Sex eFigure 10. Changes in Age-Standardized, County-Level, Firearm-Related Homicide Rates Between 2000 and 2019 Among Males, by Race and Ethnicity and Age eFigure 11. Changes in Age-Standardized, County-Level, Firearm-Related Homicide Rates Between 2000 and 2019 Among Females, by Race and Ethnicity and Age eTable 1. Counties With Age-Standardized, Homicide Rates Greater than 100 Per 100 000 Population in 2019 Among American Indian or Alaska Native Males Aged 15 to 44 Years and Black Males Aged 15 to 64 Years eTable 2. Median Changes in Age-Standardized, County-Level Homicide Rates and the Proportion of Counties That Saw Increases and Decreases Between 2000 and 2019, by Race and Ethnicity, Sex, and Age [file jamanetwopen-e2462069-s001.pdf]

Supplementary Online Content

Strassle PD, Kendrick P, Baumann MB, et al. Homicide rates across county, race, ethnicity, age, and sex in the US. *JAMA Netw Open*. 2025;8(2):e2462069.
doi:10.1001/jamanetworkopen.2024.62069

eFigure 1. Age-Standardized, County-Level Homicide Rates in 2019 by Race and Ethnicity, Sex, and Age
eFigure 2. Map of Age-Standardized, County-Level Homicide Rates in 2019 by Race and Ethnicity and Age, Males
eFigure 3. Map of Age-Standardized, County-Level Homicide Rates in 2019 by Race and Ethnicity and Age, Females
eFigure 4. Map of Age-Standardized, County-Level Changes in Homicide Rates From 2000 to 2019 by Race and Ethnicity and Age, Females
eFigure 5. Percentage of all Homicides That Were Firearm-Related in 2019, by Race and Ethnicity, Sex, and Age
eFigure 6. Age-Standardized, National Firearm-Related Homicide Rates in 2019, by Race and Ethnicity, Sex, and Age
eFigure 7. Map of Age-Standardized, County-Level Firearm-Related Homicide Rates in 2019 by Race and Ethnicity and Age, Males
eFigure 8. Map of Age-Standardized, County-Level Firearm-Related Homicide Rates in 2019 by Race and Ethnicity and Age, Females
eFigure 9. Changes in Age-Standardized, National Firearm-Related Homicide Rates From 2000 to 2019, by Race and Ethnicity, Age, and Sex
eFigure 10. Changes in Age-Standardized, County-Level, Firearm-Related Homicide Rates Between 2000 and 2019 Among Males, by Race and Ethnicity and Age
eFigure 11. Changes in Age-Standardized, County-Level, Firearm-Related Homicide Rates Between 2000 and 2019 Among Females, by Race and Ethnicity and Age
eTable 1. Counties With Age-Standardized, Homicide Rates Greater than 100 Per 100 000 Population in 2019 Among American Indian or Alaska Native Males Aged 15 to 44 Years and Black Males Aged 15 to 64 Years
eTable 2. Median Changes in Age-Standardized, County-Level Homicide Rates and the Proportion of Counties That Saw Increases and Decreases Between 2000 and 2019, by Race and Ethnicity, Sex, and Age

This supplementary material has been provided by the authors to give readers additional information about their work.

© 2025 Strassle PD et al. *JAMA Network Open*.

eFigure 1: Age-standardized, county-level homicide rates in 2019 by race and ethnicity, sex, and age.

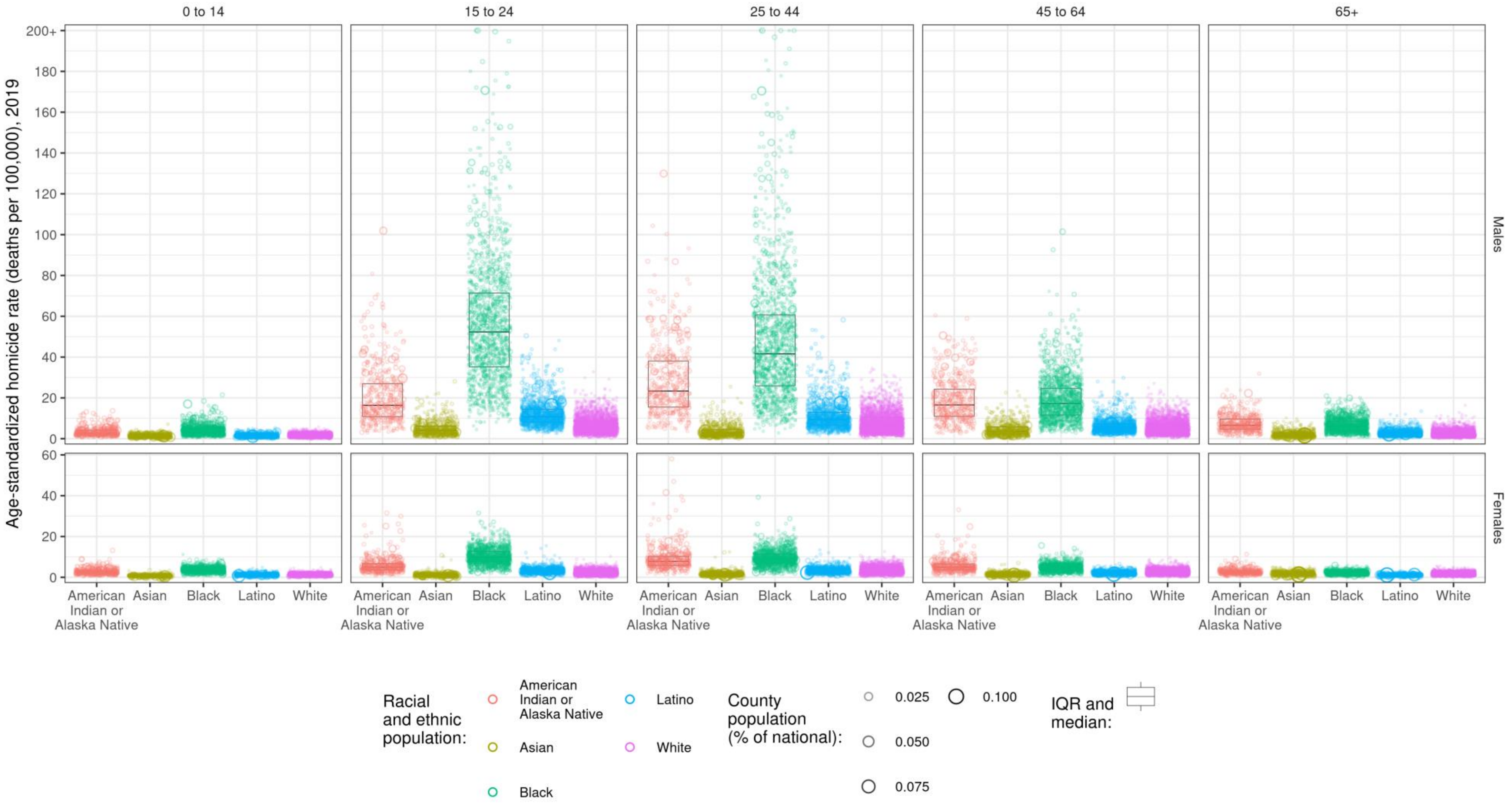

Box-and-whisker plot of county-level homicide rates in 2019 by race and ethnicity, sex, and age. Each circle represents one county in the United States. Estimates have been masked for counties with a mean annual population of <1000 people within a racial and ethnic population because model performance declined notably below this threshold. Mortality rates were capped at 200 deaths per 100,000.

© 2025 Strassle PD et al. *JAMA Network Open*.

eFigure 2: Map of age-standardized, county-level homicide rates in 2019 by race and ethnicity and age, males.

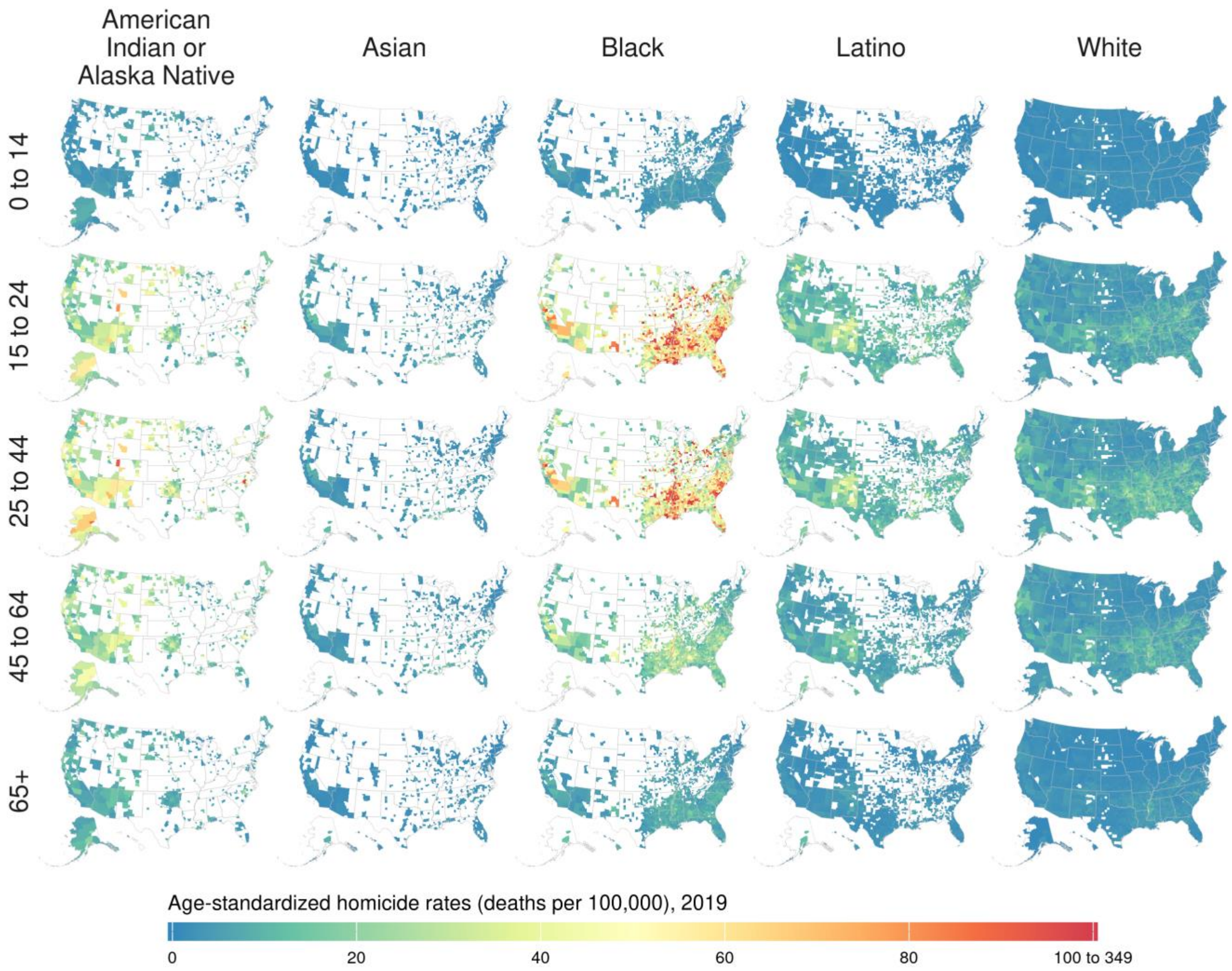

County-level homicide rates in 2019 among American Indian or Alaska Native, Asian, Black, Latino, and White males, by age. Estimates have been masked (shown in white) for counties with a mean annual population of <1000 people because model performance declined notably below this threshold

© 2025 Strassle PD et al. *JAMA Network Open*.

eFigure 3: Map of age-standardized, county-level homicide rates in 2019 by race and ethnicity and age, females.

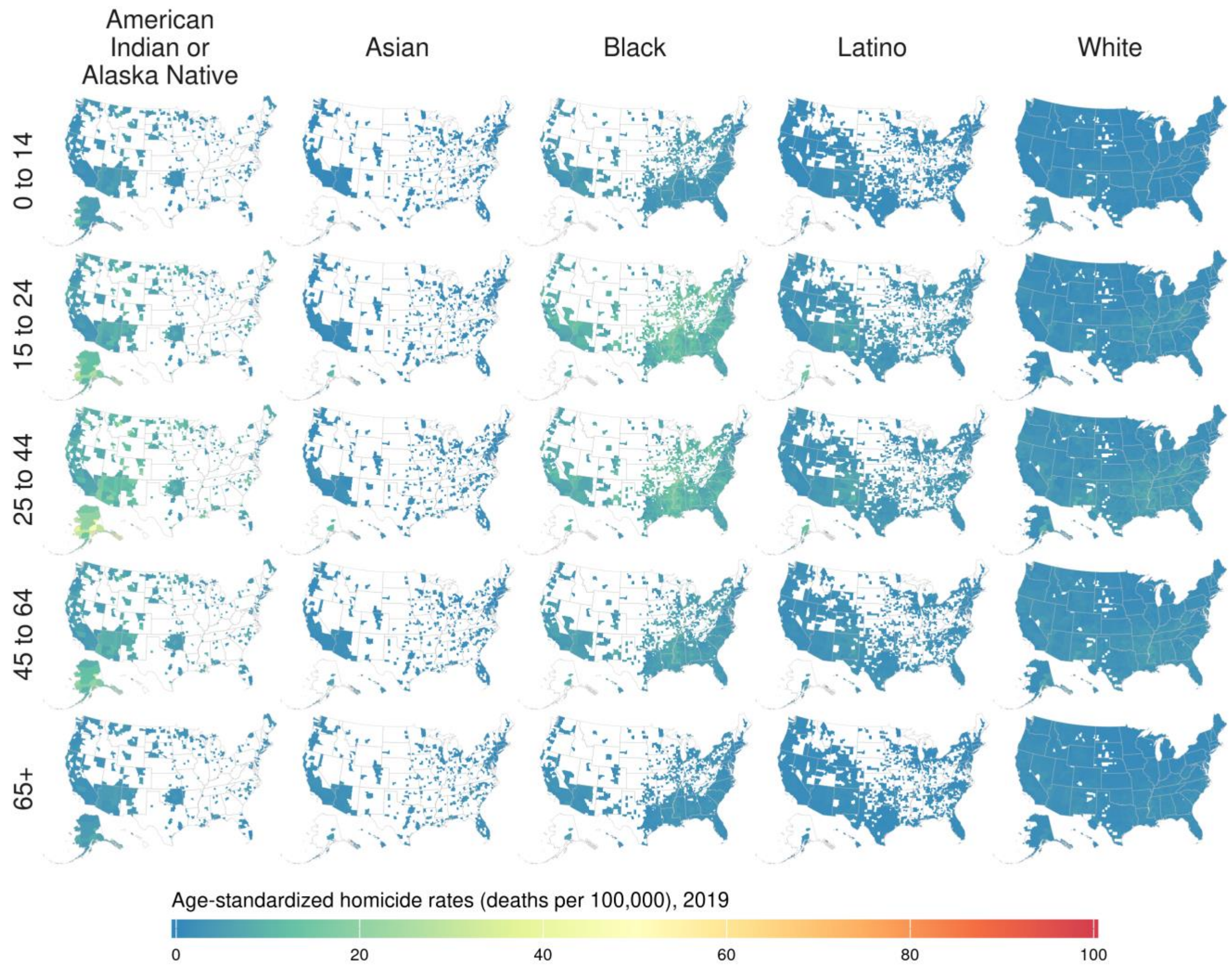

County-level homicide rates in 2019 among American Indian or Alaska Native, Asian, Black, Latino, and White females, by age. Estimates have been masked (shown in white) for counties with a mean annual population of <1000 people because model performance declined notably below this threshold

© 2025 Strassle PD et al. *JAMA Network Open*.

eFigure 4: Map of age-standardized, county-level changes in homicide rates from 2000 to 2019 by race and ethnicity and age, females.

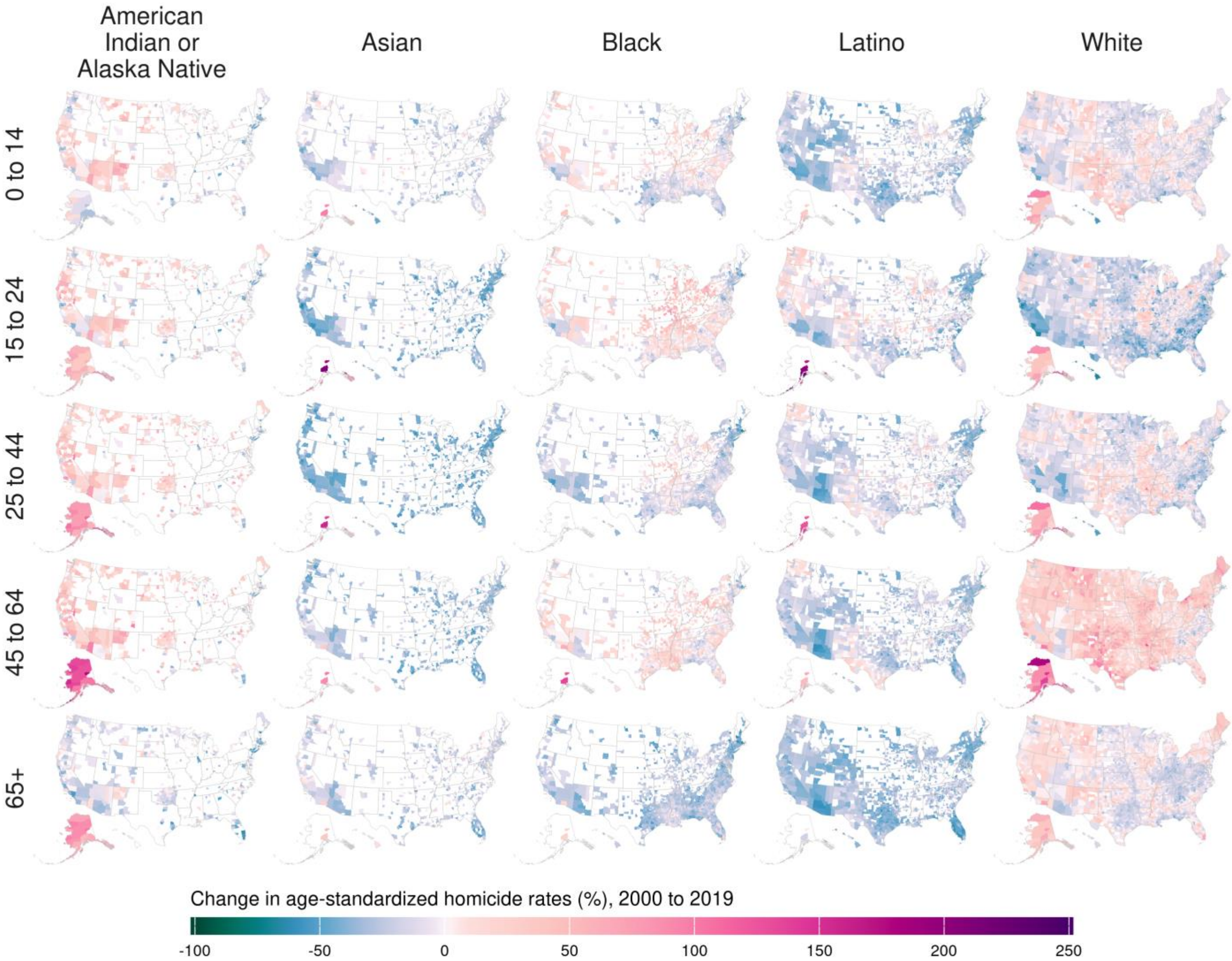

Changes in county-level homicide rates between 2000 and 2019 among females, by race and ethnicity and age. Estimates have been masked (shown in white) for counties with a mean annual population of <1000 people because model performance declined notably below this threshold.

© 2025 Strassle PD et al. *JAMA Network Open*.

eFigure 5: Percentage of all homicides that were firearm-related in 2019, by race and ethnicity, sex, and age.

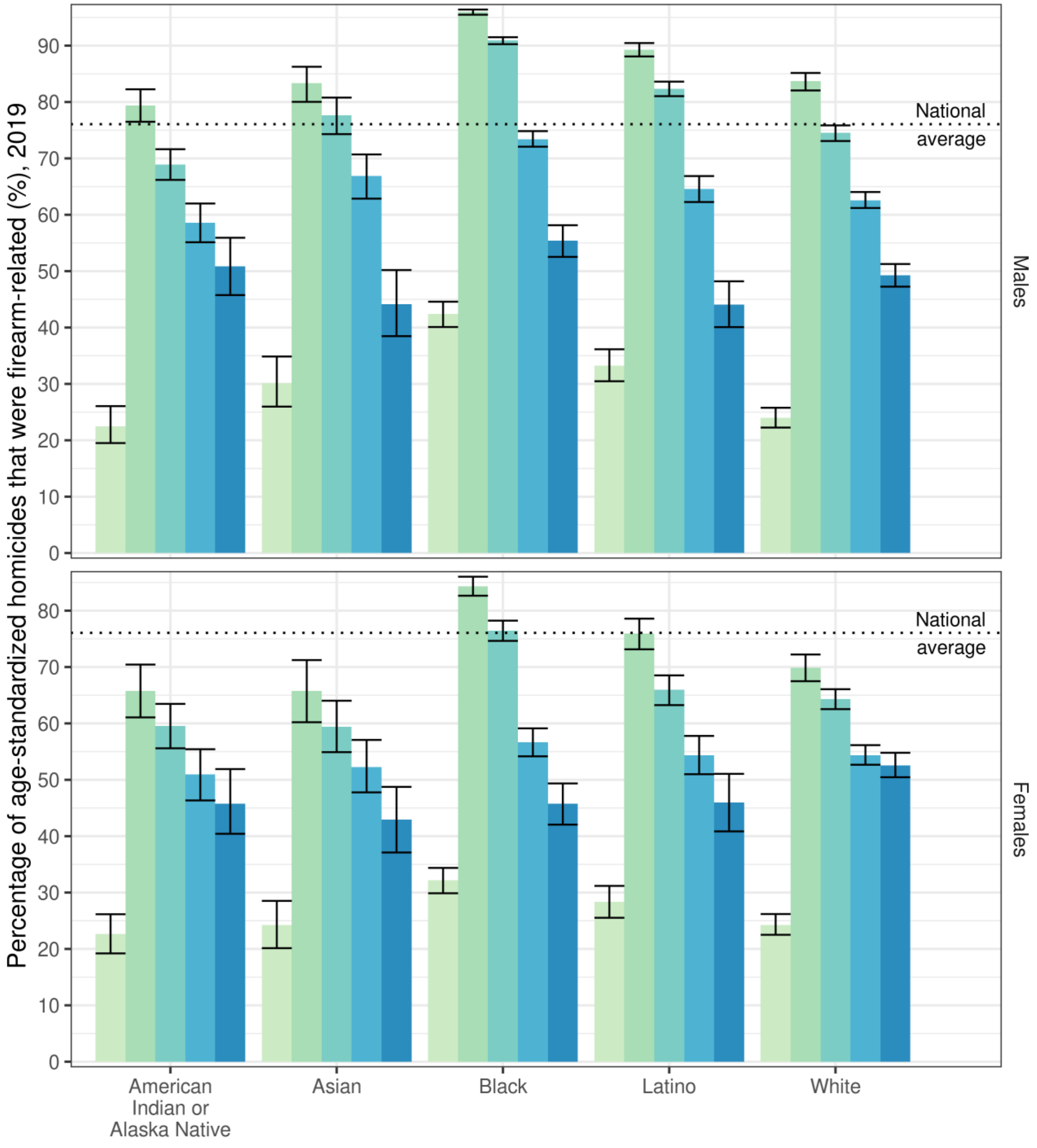

Percentage of all homicides that were firearm-related in 2019, by race and ethnicity, sex, and age. Bars represent the 95% uncertainty intervals.

© 2025 Strassle PD et al. *JAMA Network Open*.

eFigure 6: Age-standardized, national firearm-related homicide rates in 2019, by race and ethnicity, sex, and age.

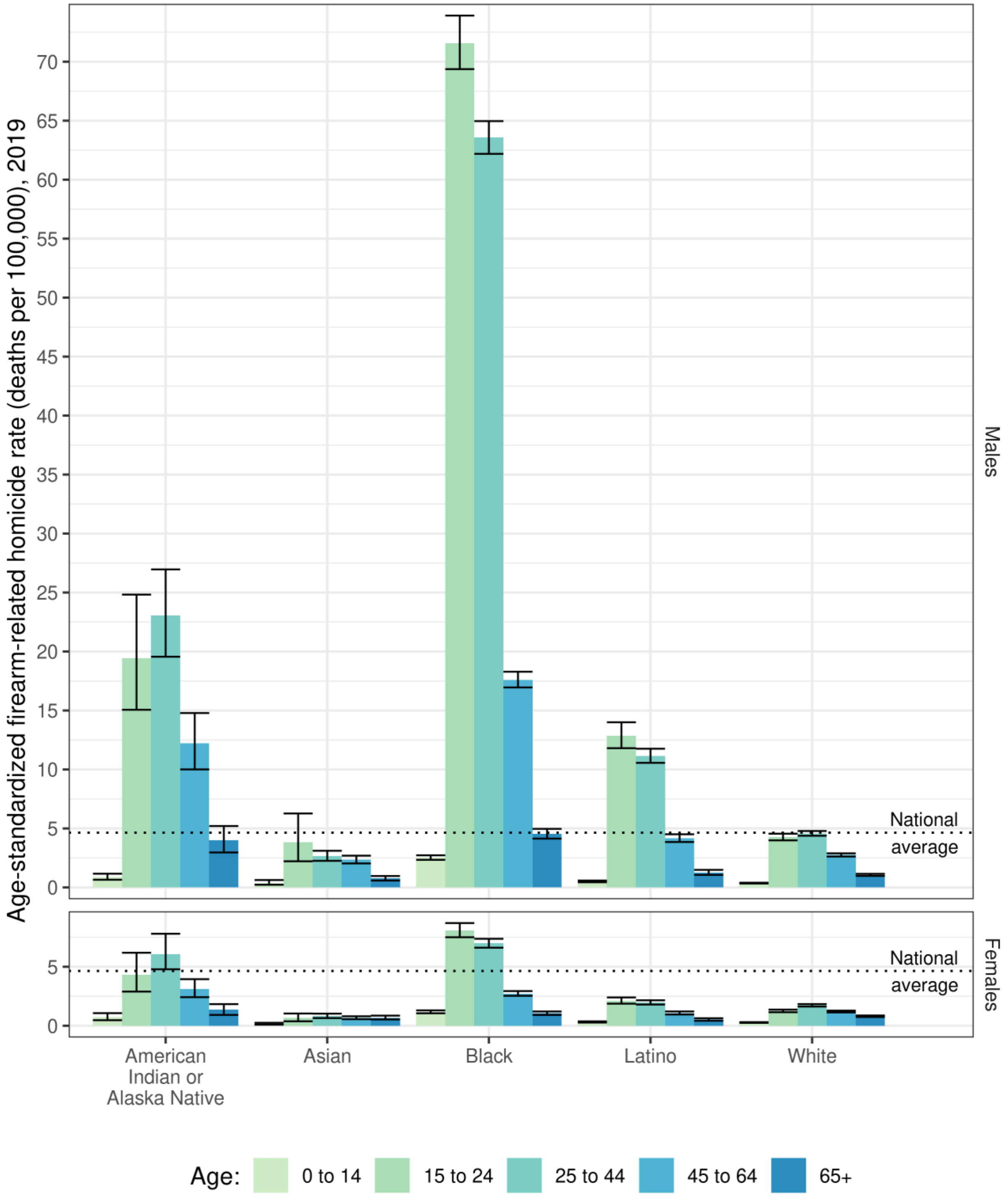

National firearm-related homicide rates in 2019, by race and ethnicity, sex, and age. Bars represent the 95% uncertainty intervals.

© 2025 Strassle PD et al. *JAMA Network Open*.

eFigure 7: Map of age-standardized, county-level firearm-related homicide rates in 2019 by race and ethnicity and age, males.

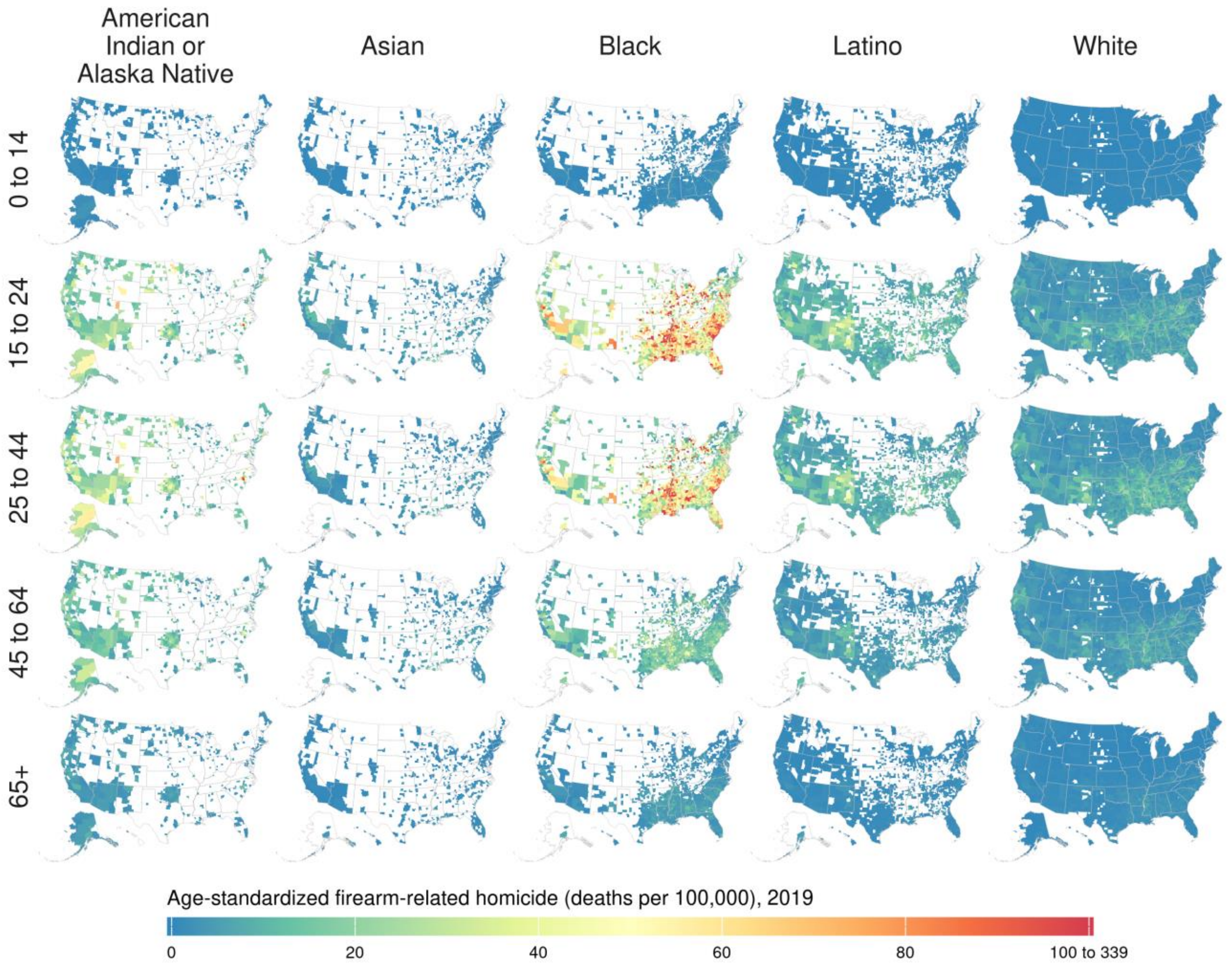

County-level firearm-related homicide rates in 2019 among American Indian or Alaska Native, Asian, Black, Latino, and White males, by age. Estimates have been masked (shown in white) for counties with a mean annual population of <1000 people because model performance declined notably below this threshold.

© 2025 Strassle PD et al. *JAMA Network Open*.

eFigure 8: Map of age-standardized, county-level firearm-related homicide rates in 2019 by race and ethnicity and age, females.

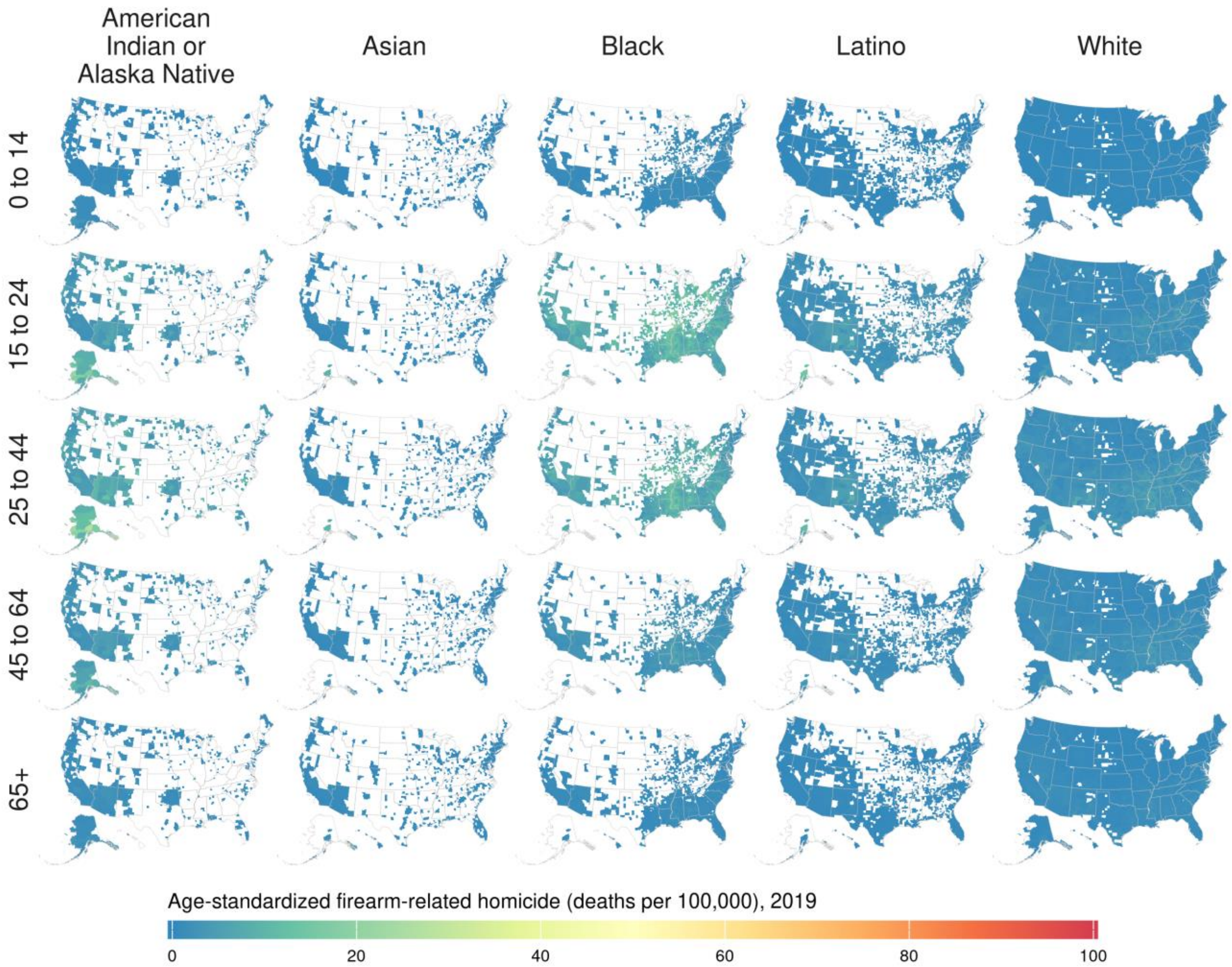

County-level firearm-related homicide rates in 2019 among American Indian or Alaska Native, Asian, Black, Latino, and White females, by age. Estimates have been masked (shown in white) for counties with a mean annual population of <1000 people because model performance declined notably below this threshold.

© 2025 Strassle PD et al. *JAMA Network Open*.

eFigure 9: Changes in age-standardized, national, firearm-related homicide rates from 2000 to 2019, by race and ethnicity, age, and sex.

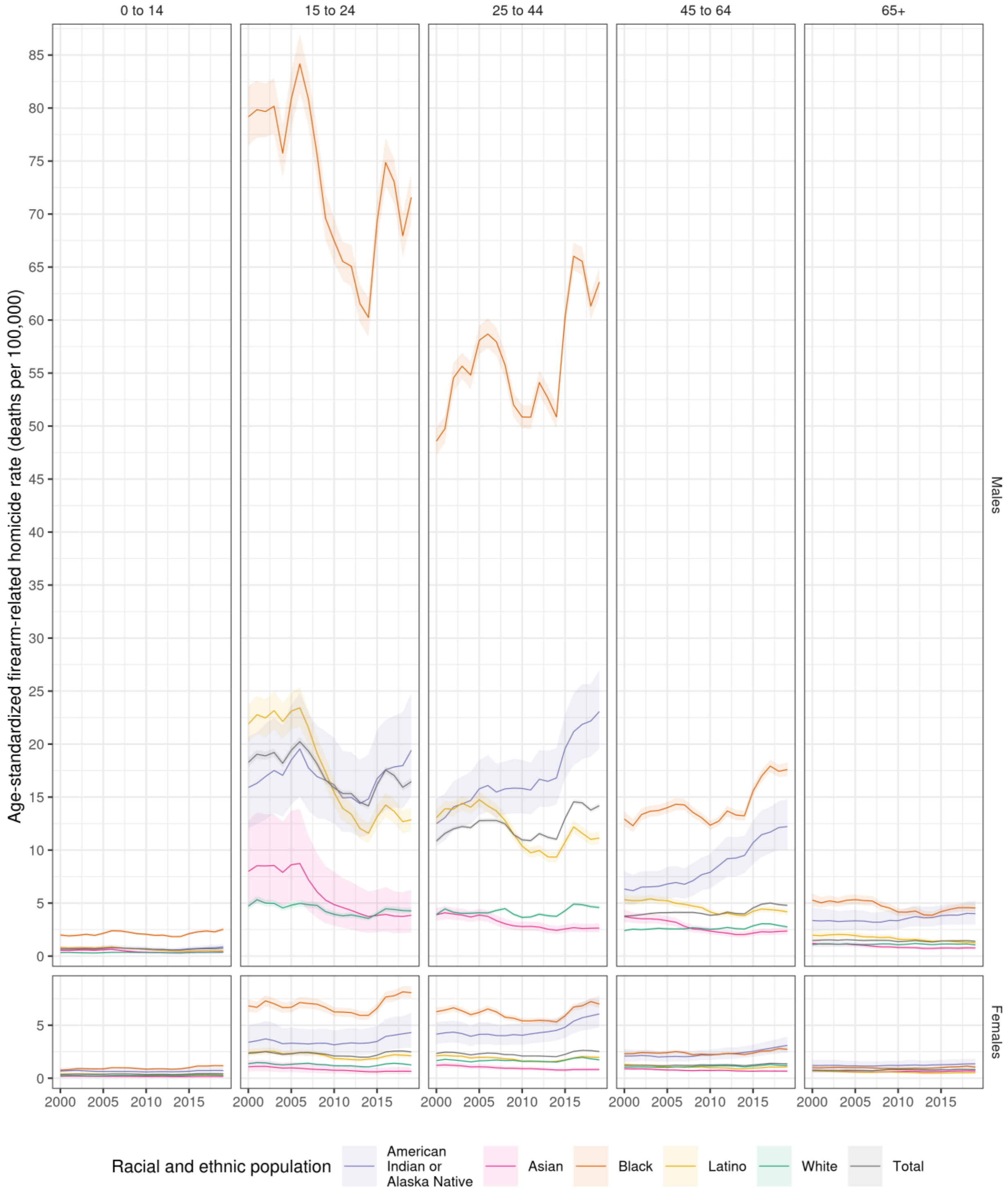

Changes in national, firearm-related homicide rates from 2000 to 2019, by race and ethnicity, age, and sex. Shaded areas indicate the 95% uncertainty intervals.

© 2025 Strassle PD et al. *JAMA Network Open*.

eFigure 10: Changes in age-standardized, county-level, firearm-related homicide rates between 2000 and 2019 among males, by race and ethnicity and age.

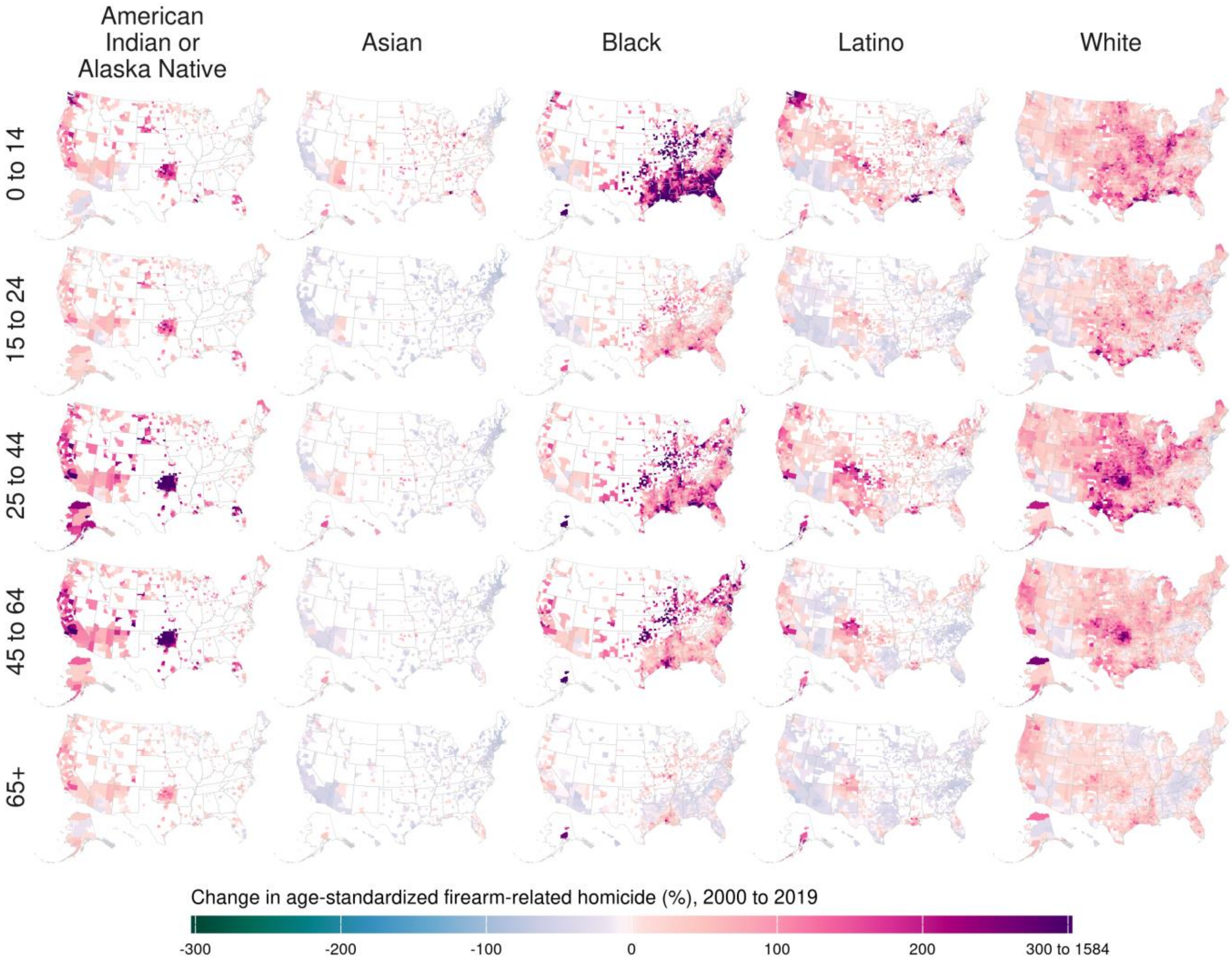

Changes in county-level, firearm-related homicide rates between 2000 and 2019 among males, by race and ethnicity and age. Estimates have been masked (shown in white) for counties with a mean annual population of <1000 people because model performance declined notably below this threshold.

© 2025 Strassle PD et al. *JAMA Network Open*.

eFigure 11: Changes in age-standardized, county-level, firearm-related homicide rates between 2000 and 2019 among females, by race and ethnicity and age.

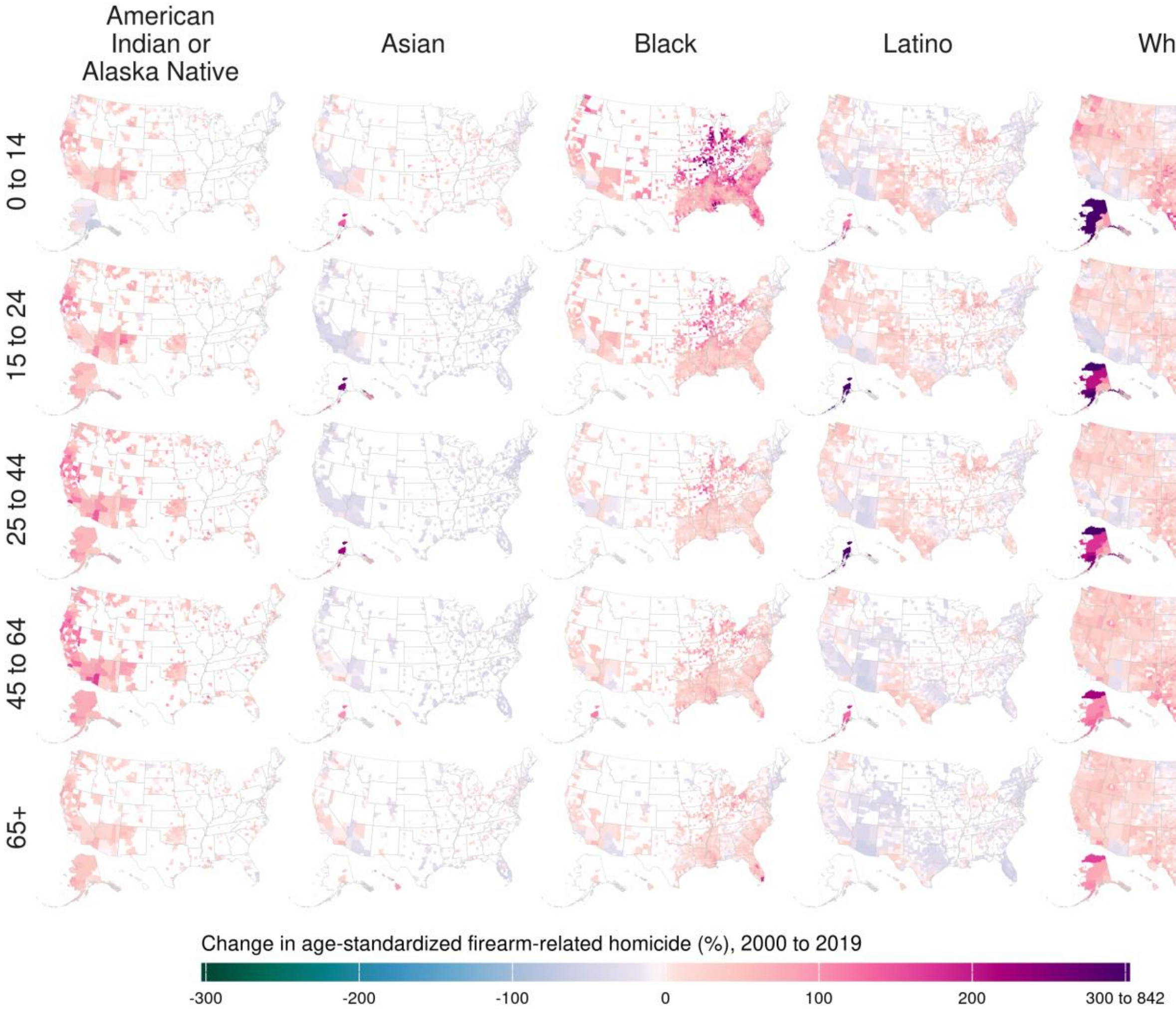

Changes in county-level, firearm-related homicide rates between 2000 and 2019 among females, by race and ethnicity and age. Estimates have been masked (shown in white) for counties with a mean annual population of <1000 people because model performance declined notably below this threshold.

© 2025 Strassle PD et al. *JAMA Network Open*.

eTable 1: Counties with age-standardized homicide rates greater than 100 per 100 000 population in 2019 among 15-44-year-old American Indian or Alaska Native males and 15-64-year-old Black males.

	American Indian or Alaska Native males		Black males		
**County or combined county-unit**	**15-24 Rate (95% UI) *Pop. size***	**25-44 Rate (95% UI) *Pop. size***	**15-24 Rate (95% UI) *Pop. size***	**25-44 Rate (95% UI) *Pop. size***	**45-64 Rate (95% UI) *Pop. size***
**Alabama**					
Butler County				111.2 (80.6-149.1) *1,011*	
Dallas County			105.9 (84.8-130.6) *1,962*	142.9 (115.1-177.3) *2,874*	
Jefferson County			152.9 (139.4-166.8) *20,267*	167.6 (151.3-184.1) *37,408*	
Lowndes County				138.2 (103.8-183.9) *829*	
Macon County			115.1 (93.2-140.8) *1,497*	148.6 (117.3-186.8) *1,403*	
Mobile County			100.1 (87.3-114.2) *11,192*	112.5 (97.8-129.9) *19,056*	
**Arkansas**					
Ashley County			104.8 (72.9-141.3) *318*		
Bradley County			112.1 (74.6-157.7) *146*		
Cleveland County			102.0 (63.1-155.6) *57*		
Crittenden County			117.5 (98.3-138.2) *2,002*	117.7 (94.4-143.6) *3,043*	
Desha County			117.5 (84.2-157.4) *335*	108.0 (76.2-149.3) *507*	
Garland County			122.7 (91.4-159.4) *829*	118.3 (81.7-161.4) *1,154*	
Jefferson County			117.6 (99.7-137.5) 3,286	142.7 (118.7-169.2) *4,925*	
Mississippi County			142.5 (117.1-169.7) *1,113*	161.9 (127.7-198.7) *1,710*	

© 2025 Strassle PD et al. *JAMA Network Open*.

	American Indian or Alaska Native males		Black males		
**County or combined county-unit**	**15-24**<br>**Rate (95% UI)**<br>***Pop. size***	**25-44**<br>**Rate (95% UI)**<br>***Pop. size***	**15-24**<br>**Rate (95% UI)**<br>***Pop. size***	**25-44**<br>**Rate (95% UI)**<br>***Pop. size***	**45-64**<br>**Rate (95% UI)**<br>***Pop. size***
Phillips County			168.9 (137.0-205.2)<br>*778*	233.9 (184.8-285.8)<br>*978*	
Poinsett County			102.4 (63.4-153.7)<br>*142*		
**District of Columbia**			129.8 (119.3-141.5)<br>*16,542*	158.7 (144.2-174.4)<br>*42,117*	
**Florida**					
Duval County			116.3 (104.1-128.8)<br>*21,823*		
Manatee County			102.8 (80.5-126.0)<br>*2,759*		
**Georgia**					
Colquitt County			100.7 (77.0-129.5)<br>*865*		
Muscogee County			103.6 (88.3-120.4)<br>*6,801*		
**Illinois**					
Cook County			170.7 (161.5-180.2)<br>81,684	170.4 (161.5-179.5)<br>*152,211*	
Kankakee County			110.7 (85.9-138.8)<br>*1,530*		
Macon County			105.3 (84.5-127.8)<br>*1,679*		
Madison County			118.7 (96.2-142.7)<br>*2,063*	112.2 (86.1-138.9)<br>*2,970*	
Peoria County			138.0 (114.8-163.7)<br>*2,976*	143.0 (115.6-175.0)<br>*4,209*	
Saint Clair County			172.6 (151.7-194.2)<br>*6,073*	183.9 (160.4-209.2)<br>*9,520*	
Sangamon County			110.9 (89.5-134.0)<br>*2,401*		
Vermilion County			142.0 (113.1-174.1)<br>*981*	118.4 (91.3-147.0)<br>*1,858*	

© 2025 Strassle PD et al. *JAMA Network Open*.

	American Indian or Alaska Native males		Black males		
County or combined county-unit	15-24<br>Rate (95% UI)<br>*Pop. size*	25-44<br>Rate (95% UI)<br>*Pop. size*	15-24<br>Rate (95% UI)<br>*Pop. size*	25-44<br>Rate (95% UI)<br>*Pop. size*	45-64<br>Rate (95% UI)<br>*Pop. size*
Winnebago County			146.9 (124.7-172.5)<br>*3,377*	116.4 (94.2-140.8)<br>*5,116*	
**Indiana**					
Allen County			179.0 (154.9-206.3)<br>*4,120*	164.0 (137.1-194.9)<br>*6,008*	
Elkhart County			124.8 (97.6-154.4)<br>*1,195*	108.3 (82.6-140.2)<br>*1,733*	
Lake County			194.8 (174.2-216.9)<br>*8,449*	191.1 (167.9-215.4)<br>*12,661*	
Marion County			152.7 (139.0-166.6)<br>*20,989*	157.4 (141.0-173.7)<br>*36,822*	
Saint Joseph County			131.4 (108.9-156.2)<br>*3,423*	114.6 (92.7-141.6)<br>*4,938*	
Vanderburgh County			109.0 (82.3-137.7)<br>*1,756*		
**Kansas**					
Sedgwick County			105.0 (85.5-126.9)<br>*4,124*		
Shawnee County			152.0 (119.4-188.2)<br>*1,324*	149.9 (112.6-194.4)<br>*1,977*	
Wyandotte County			172.4 (143.2-203.0)<br>*2,408*	202.3 (165.5-240.1)<br>*4,256*	
**Kentucky**					
Jefferson County			152.0 (136.8-167.4)<br>*12,893*	149.4 (133.4-166.8)<br>*22,286*	
**Louisiana**					
Acadia Parish			154.2 (120.1-191.5)<br>*895*	150.9 (114.7-194.0)<br>*1,438*	
Caddo Parish			107.6 (94.2-121.9)<br>*8,511*	109.4 (94.7-125.0)<br>*15,214*	
East Baton Rouge Parish			132.9 (119.5-146.2)<br>*18,002*	131.9 (118.0-147.0)<br>*26,497*	

© 2025 Strassle PD et al. *JAMA Network Open*.

	American Indian or Alaska Native males		Black males		
County or combined county-unit	15-24<br>Rate (95% UI)<br>*Pop. size*	25-44<br>Rate (95% UI)<br>*Pop. size*	15-24<br>Rate (95% UI)<br>*Pop. size*	25-44<br>Rate (95% UI)<br>*Pop. size*	45-64<br>Rate (95% UI)<br>*Pop. size*
Jefferson Davis Parish			136.0 (99.5-176.4)<br>*441*	121.4 (83.8-162.4)<br>*718*	
Jefferson Parish			121.1 (105.8-136.4)<br>*8,204*	112.4 (96.6-129.6)<br>*15,577*	
Livingston Parish			105.4 (74.9-142.8)<br>*749*		
Orleans Parish			136.0 (123.1-150.1)<br>*15,172*	159.2 (142.9-176.7)<br>*29,046*	
Rapides Parish			101.2 (82.8-120.6)<br>*3,300*		
Saint John the Baptist Parish			114.3 (92.4-138.3)<br>*1,827*	118.5 (93.7-147.1)<br>*3,164*	
Terrebonne Parish			106.8 (80.9-135.7)<br>*1,697*		
Vermilion Parish			132.0 (97.6-172.3)<br>*630*	132.0 (94.1-178.6)<br>*1,040*	
**Maryland**					
Baltimore City			203.0 (188.5-217.4)<br>*24,541*	323.0 (300.6-346.7)<br>*49,496*	101.5 (85.7-116.4)<br>*42,791*
Baltimore County			101.2 (90.9-111.4)<br>*17,840*	104.7 (92.3-117.5)<br>*32,472*	
**Michigan**					
Genesee County			172.1 (149.5-194.7)<br>*6,355*	149.8 (127.9-173.3)<br>*9,375*	
Saginaw County			140.3 (116.0-167.1)<br>*2,980*	129.3 (103.0-159.8)<br>*4,321*	
Wayne County			110.1 (100.9-120.4)<br>*45,264*	127.5 (116.6-138.6)<br>*80,540*	
**Mississippi**					
Adams County				141.2 (108.0-179.5)<br>*1,799*	
Alcorn County			102.3 (70.3-140.3)<br>*364*		

© 2025 Strassle PD et al. *JAMA Network Open*.

	American Indian or Alaska Native males		Black males		
County or combined county-unit	15-24<br>Rate (95% UI)<br>*Pop. size*	25-44<br>Rate (95% UI)<br>*Pop. size*	15-24<br>Rate (95% UI)<br>*Pop. size*	25-44<br>Rate (95% UI)<br>*Pop. size*	45-64<br>Rate (95% UI)<br>*Pop. size*
Attala County			111.8 (83.5-141.9)<br>*530*	110.3 (82.1-142.7)<br>*884*	
Bolivar County			103.6 (82.3-128.6)<br>*1,382*	130.9 (103.0-163.7)<br>*2,209*	
Coahoma County			138.9 (111.9-167.9)<br>*1,391*	177.8 (142.1-215.4)<br>*1,773*	
Grenada County			151.3 (116.6-191.7)<br>*566*	142.9 (109.3-184.1)<br>*1,079*	
Hinds County			106.2 (94.8-117.9)<br>*13,045*	131.2 (115.8-146.5)<br>*21,492*	
Holmes County			104.1 (79.2-131.9)<br>*1,073*	123.7 (93.7-155.6)<br>*1,633*	
Humphreys County				105.7 (73.9-142.0)<br>*647*	
Jefferson Davis County				118.0 (82.7-161.3)<br>*766*	
Leflore County				130.4 (101.7-161.2)<br>*2,388*	
Lincoln County			101.5 (75.5-132.1)<br>*667*		
Sharkey County				101.2 (65.2-145.9)<br>*280*	
Tunica County				105.8 (78.1-138.1)<br>*860*	
Washington County			141.6 (115.2-167.2)<br>*2,104*	180.7 (149.5-214.0)<br>*3,484*	
**Missouri**					
Cape Girardeau County			103.0 (72.6-135.8)<br>*762*		
Jackson County			184.8 (167.3-202.4)<br>*11,758*	236.7 (214.1-261.2)<br>*20,783*	
Pemiscot County			107.9 (73.0-149.6)<br>*310*	109.0 (72.1-158.4)<br>*409*	

© 2025 Strassle PD et al. *JAMA Network Open*.

	American Indian or Alaska Native males		Black males		
County or combined county-unit	15-24<br>Rate (95% UI)<br>*Pop. size*	25-44<br>Rate (95% UI)<br>*Pop. size*	15-24<br>Rate (95% UI)<br>*Pop. size*	25-44<br>Rate (95% UI)<br>*Pop. size*	45-64<br>Rate (95% UI)<br>*Pop. size*
Saint Louis City			235.0 (212.3-259.0)<br>*9,195*	348.9 (313.2-385.5)<br>*16,821*	
Saint Louis County			199.5 (182.1-216.9)<br>*18,326*	196.8 (177.9-216.6)<br>*29,739*	
Scott County			130.6 (92.0-175.6)<br>*352*	113.0 (75.8-162.7)<br>*580*	
**New Jersey**					
Salem County			104.5 (74.1-139.0)<br>*717*		
**New York**					
Onondaga County			113.6 (93.8-135.4)<br>*4,942*		
**North Carolina**					
Cumberland County		100.2 (56.2-166.2)<br>*694*			
Hoke County		104.3 (58.4-165.9)<br>*589*			
Lee County			142.5 (104.4-187.8)<br>*810*	124.2 (89.4-167.0)<br>*1,454*	
New Hanover County			106.3 (78.9-136.3)<br>*2,467*	101.2 (73.9-134.5)<br>*3,497*	
Robeson County	101.9 (74.2-131.7)<br>*3,828*	129.9 (103.3-156.3)<br>*6,370*	123.1 (93.0-157.3)<br>*2,541*		
Rockingham County			107.5 (82.9-136.8)<br>*1,046*		
Scotland County			101.7 (77.4-130.2)<br>*939*		
Vance County			133.2 (103.3-167.1)<br>*1,539*	140.5 (106.0-178.2)<br>*2,301*	
**Ohio**					
Clark County			115.9 (87.2-149.3)<br>*1,139*	106.5 (76.5-143.5)<br>*1,537*	

© 2025 Strassle PD et al. *JAMA Network Open*.

	American Indian or Alaska Native males		Black males		
County or combined county-unit	15-24<br>Rate (95% UI)<br>*Pop. size*	25-44<br>Rate (95% UI)<br>*Pop. size*	15-24<br>Rate (95% UI)<br>*Pop. size*	25-44<br>Rate (95% UI)<br>*Pop. size*	45-64<br>Rate (95% UI)<br>*Pop. size*
Cuyahoga County			132.2 (121.0-144.2)<br>*26,440*	134.7 (122.0-149.2)<br>*42,484*	
Hamilton County			134.2 (120.5-148.4)<br>*15,895*	111.8 (98.7-126.1)<br>*25,961*	
Jefferson County			123.6 (85.3-167.7)<br>*356*		
Lucas County			100.5 (85.8-117.0)<br>*6,954*		
Mahoning County			175.5 (147.5-203.4)<br>*3,095*	159.9 (132.2-191.6)<br>*4,445*	
Montgomery County			134.3 (117.1-153.4)<br>*8,803*	128.5 (110.3-149.7)<br>*13,625*	
Summit County			130.3 (112.4-150.4)<br>*6,677*	122.8 (103.6-143.8)<br>*9,760*	
Trumbull County			128.4 (102.2-156.9)<br>*1,512*	109.1 (83.9-140.5)<br>*2,349*	
**Oklahoma**					
Choctaw County			130.6 (85.5-192.1)<br>*117*	112.9 (66.2-172.6)<br>*173*	
McCurtain County			121.7 (81.7-174.4)<br>*213*	102.5 (66.4-147.5)<br>*345*	
Oklahoma County			102.1 (89.6-116.3)<br>*9,899*	109.4 (93.1-126.3)<br>*17,664*	
Tulsa County			127.8 (108.7-147.7)<br>*6,069*	138.7 (115.3-164.3)<br>*9,532*	
**Pennsylvania**					
Allegheny County			155.4 (139.2-172.4)<br>*13,092*	148.0 (129.9-166.2)<br>*21,454*	
Beaver County			103.5 (77.3-131.0)<br>*947*		
Dauphin County			101.1 (85.3-119.3)<br>*3,952*		

© 2025 Strassle PD et al. *JAMA Network Open*.

	American Indian or Alaska Native males		Black males		
County or combined county-unit	15-24<br>Rate (95% UI)<br>*Pop. size*	25-44<br>Rate (95% UI)<br>*Pop. size*	15-24<br>Rate (95% UI)<br>*Pop. size*	25-44<br>Rate (95% UI)<br>*Pop. size*	45-64<br>Rate (95% UI)<br>*Pop. size*
Delaware County			104.9 (91.9-119.5)<br>*10,631*		
Lawrence County			124.2 (88.5-169.3)<br>*421*	108.1 (68.0-159.2)<br>*536*	
Philadelphia County			135.4 (126.3-145.1)<br>*44,943*	145.2 (134.6-156.5)<br>*85,235*	
York County			112.3 (90.6-135.8)<br>*2,590*		
**South Carolina**					
Barnwell County			119.2 (91.2-154.4)<br>*624*	115.2 (83.1-155.0)<br>*1,024*	
Beaufort County			108.1 (84.3-134.3)<br>*2,603*		
Charleston County			144.7 (126.2-165.1)<br>*6,970*	125.7 (106.5-144.8)<br>*13,983*	
Colleton County			159.3 (124.2-195.8)<br>*864*	146.3 (110.6-186.4)<br>*1,566*	
Dillon County			100.1 (76.6-128.3)<br>*1,054*	100.7 (76.7-129.5)<br>*1,756*	
**Tennessee**					
Davidson County			105.1 (94.0-117.1)<br>*14,331*		
Gibson County			108.4 (77.5-141.9)<br>*639*		
Hamilton County			109.1 (92.9-125.3)<br>*5,330*	100.0 (83.3-117.1)<br>*8,571*	
Knox County			113.5 (95.3-132.4)<br>*4,257*	109.1 (88.6-132.3)<br>*5,317*	
Madison County				100.5 (79.2-124.1)<br>*4,107*	
Robertson County			120.4 (84.7-163.4)<br>*371*		

© 2025 Strassle PD et al. *JAMA Network Open*.

	American Indian or Alaska Native males		Black males		
County or combined county-unit	15-24<br>Rate (95% UI)<br>*Pop. size*	25-44<br>Rate (95% UI)<br>*Pop. size*	15-24<br>Rate (95% UI)<br>*Pop. size*	25-44<br>Rate (95% UI)<br>*Pop. size*	45-64<br>Rate (95% UI)<br>*Pop. size*
Shelby County			131.3 (120.7-142.0)<br>*37,182*	128.0 (117.1-139.2)<br>*63,316*	
**Texas**					
Hardin County			108.0 (67.3-157.2)<br>*254*		
Harrison County			102.6 (76.8-131.5)<br>*1,044*		
**Virginia**					
Hopewell City			123.0 (88.3-164.8)<br>*690*	112.3 (73.6-164.0)<br>*1,189*	
Martinsville City			123.9 (72.7-185.3)<br>*378*		
Petersburg City			180.1 (148.2-219.6)<br>*1,294*	185.4 (146.1-232.5)<br>*2,880*	
Pittsylvania County/Danville City			101.7 (83.8-122.7)<br>*2,231*		
Portsmouth City				102.2 (81.9-123.8)<br>*6,697*	
Richmond City			111.9 (94.5-130.0)<br>*6,935*	121.4 (99.5-142.6)<br>*13,956*	
**West Virginia**					
Cabell County			127.1 (91.9-171.8)<br>*525*	109.3 (71.4-157.6)<br>*679*	
Kanawha County			118.8 (92.5-147.7)<br>*1,384*	103.4 (75.5-134.9)<br>*2,016*	
**Wisconsin**					
Milwaukee County			120.9 (108.5-133.8)<br>*19,890*	139.5 (125.8-155.9)<br>*31,395*	

Counties are organized alphabetically by state and then county. Average population sizes were rounded for interpretation. UI=uncertainty interval. Pop=population.

© 2025 Strassle PD et al. *JAMA Network Open*.

eTable 2: Median changes in age-standardized, county-level homicide rates and the proportion of counties that saw increases and decreases between 2000 and 2019, by race and ethnicity, sex, and age.

Racial and ethnic population	Number of counties	Age	Males					Females				
			% Change median (IQR)	Counties with increases (%)		Counties with decreases (%)		% Change Median (IQR)	Counties with increases (%)		Counties with decreases (%)	
				Total[a]	Stat. Sig.[b]	Total[a]	Stat. Sig.[b]		Total[a]	Stat. Sig.[b]	Total[a]	Stat. Sig.[b]
American Indian or Alaska Native	474	0 to 14	19.6 (−6.9, 43.5)	318 (67.1%)	1 (0.2%)	156 (32.9%)	0 (0.0%)	−0.1 (−15.1, 13.1)	236 (49.8%)	0 (0.0%)	238 (50.2%)	0 (0.0%)
		15 to 24	8.5 (−17.4, 38.4)	285 (60.1%)	11 (2.3%)	189 (39.9%)	6 (1.3%)	4.8 (−14.6, 21.6)	273 (57.6%)	0 (0.0%)	201 (42.4%)	3 (0.6%)
		25 to 44	53.8 (21.6, 107.1)	423 (89.2%)	87 (18.4%)	51 (10.8%)	0 (0.0%)	16.2 (1.0, 33.4)	360 (75.9%)	2 (0.4%)	114 (24.1%)	0 (0.0%)
		45 to 64	43.0 (16.3, 94.5)	412 (86.9%)	53 (11.2%)	62 (13.1%)	0 (0.0%)	18.4 (2.6, 35.0)	371 (78.3%)	3 (0.6%)	103 (21.7%)	0 (0.0%)
		65+	2.7 (−15.5, 23.7)	255 (53.8%)	0 (0.0%)	219 (46.2%)	0 (0.0%)	−15.2 (−27.1, −2.9)	102 (21.5%)	0 (0.0%)	372 (78.5%)	1 (0.2%)
Asian	667	0 to 14	5.6 (−12.3, 25.7)	396 (59.4%)	1 (0.1%)	271 (40.6%)	2 (0.3%)	−16.9 (−25.4, −7.8)	68 (10.2%)	0 (0.0%)	599 (89.8%)	1 (0.1%)
		15 to 24	−44.2 (−57.2, −32.0)	17 (2.5%)	0 (0.0%)	650 (97.5%)	131 (19.6%)	−35.9 (−45.1, −24.9)	20 (3.0%)	0 (0.0%)	647 (97.0%)	20 (3%)
		25 to 44	−31.0 (−44.9, −14.1)	74 (11.1%)	1 (0.1%)	593 (88.9%)	61 (9.1%)	−41.4 (−47.8, −34.0)	8 (1.2%)	0 (0.0%)	659 (98.8%)	34 (5.1%)
		45 to 64	−33.0 (−46.1, −20.4)	41 (6.1%)	0 (0.0%)	626 (93.9%)	41 (6.1%)	−33.8 (−41.1, −26.0)	15 (2.2%)	0 (0.0%)	652 (97.8%)	2 (0.3%)
		65+	−34.6 (−46.0, −23.5)	28 (4.2%)	0 (0.0%)	639 (95.8%)	4 (0.6%)	−17.8 (−26.2, −7.8)	70 (10.5%)	0 (0.0%)	597 (89.5%)	0 (0.0%)
Black	1488	0 to 14	42.3 (16.1, 68.7)	1302 (87.5%)	173 (11.6%)	186 (12.5%)	17 (1.1%)	−3.0 (−14.6, 6.7)	620 (41.7%)	1 (0.1%)	868 (58.3%)	7 (0.5%)
		15 to 24	24.0 (3.1, 47.8)	1158 (77.8%)	298 (20.0%)	330 (22.2%)	74 (5.0%)	7.9 (−5.5, 24.5)	983 (66.1%)	9 (0.6%)	505 (33.9%)	22 (1.5%)
		25 to 44	68.1 (39.4, 105.7)	1406 (94.5%)	729 (49.0%)	82 (5.5%)	19 (1.3%)	−12.5 (−22.9, −0.8)	342 (23.0%)	2 (0.1%)	1146 (77.0%)	34 (2.3%)
		45 to 64	30.4 (8.8, 59.9)	1249 (83.9%)	114 (7.7%)	239 (16.1%)	7 (0.5%)	5.7 (−4.8, 17.5)	953 (64.0%)	2 (0.1%)	535 (36.0%)	3 (0.2%)

© 2025 Strassle PD et al. *JAMA Network Open*.

Racial and ethnic population	Number of counties	Age	Males					Females				
			% Change median (IQR)	Counties with increases (%)		Counties with decreases (%)		% Change Median (IQR)	Counties with increases (%)		Counties with decreases (%)	
				Total[a]	Stat. Sig.[b]	Total[a]	Stat. Sig.[b]		Total[a]	Stat. Sig.[b]	Total[a]	Stat. Sig.[b]
		65+	−27.1 (−37.4, −13.9)	151 (10.1%)	1 (0.1%)	1337 (89.9%)	13 (0.9%)	−26.6 (−34.1, −19.4)	28 (1.9%)	0 (0.0%)	1460 (98.1%)	8 (0.5%)
Latino	1478	0 to 14	2.7 (−13.7, 24.2)	809 (54.7%)	5 (0.3%)	669 (45.3%)	22 (1.5%)	−24.2 (−34.5, −13.3)	111 (7.5%)	0 (0.0%)	1367 (92.5%)	22 (1.5%)
		15 to 24	−29.0 (−42.8, −14.8)	148 (10.0%)	1 (0.1%)	1330 (90.0%)	208 (14.1%)	−13.9 (−28.5, 0.6)	383 (25.9%)	0 (0.0%)	1095 (74.1%)	32 (2.2%)
		25 to 44	−3.1 (−24.3, 18.6)	690 (46.7%)	18 (1.2%)	788 (53.3%)	73 (4.9%)	−16.3 (−27.2, −4.7)	245 (16.6%)	0 (0.0%)	1233 (83.4%)	29 (2.0%)
		45 to 64	−23.4 (−38.9, −5.1)	291 (19.7%)	4 (0.3%)	1187 (80.3%)	75 (5.1%)	−22.1 (−31.1, −11.3)	140 (9.5%)	0 (0.0%)	1338 (90.5%)	8 (0.5%)
		65+	−38.9 (−47.1, −28.5)	50 (3.4%)	0 (0.0%)	1428 (96.6%)	18 (1.2%)	−32.4 (−40.1, −24.5)	8 (0.5%)	0 (0.0%)	1470 (99.5%)	3 (0.2%)
White	3051	0 to 14	3.4 (−10.5, 16.8)	1726 (56.6%)	4 (0.1%)	1325 (43.4%)	45 (1.5%)	−3.6 (−12.2, 5.9)	1207 (39.6%)	0 (0.0%)	1844 (60.4%)	7 (0.2%)
		15 to 24	2.8 (−17.0, 24.4)	1631 (53.5%)	16 (0.5%)	1420 (46.5%)	72 (2.4%)	−14.3 (−27.1, −1.9)	664 (21.8%)	1 (0.0%)	2387 (78.2%)	85 (2.8%)
		25 to 44	38.2 (12.0, 65.2)	2571 (84.3%)	279 (9.1%)	480 (15.7%)	30 (1.0%)	−5.8 (−15.6, 5.3)	1084 (35.5%)	1 (0.0%)	1967 (64.5%)	34 (1.1%)
		45 to 64	23.6 (3.8, 44.1)	2415 (79.2%)	80 (2.6%)	636 (20.8%)	15 (0.5%)	19.6 (7.0, 34.8)	2619 (85.8%)	9 (0.3%)	432 (14.2%)	1 (0.0%)
		65+	−8.4 (−20.2, 3.3)	943 (30.9%)	0 (0.0%)	2108 (69.1%)	9 (0.3%)	−5.3 (−14.8, 5.4)	1120 (36.7%)	0 (0.0%)	1931 (63.3%)	0 (0.0%)

IQR=Interquartile range. Stat Sig=Statistically significant.

© 2025 Strassle PD et al. *JAMA Network Open*.
