# Supplementary material for: Homicide Rates Across County, Race, Ethnicity, Age, and Sex in the US: A Global Burden of Disease Study
Source: JAMA Netw Open. 2025 Feb 27;8(2):e2462069. doi: 10.1001/jamanetworkopen.2024.62069 (PMC11868975; doi:10.1001/jamanetworkopen.2024.62069)
Supplement: Supplement 2. — Data Sharing Statement [file jamanetwopen-e2462069-s002.pdf]

Data Sharing Statement

Strassle. Homicide Rates Across County, Race, Ethnicity, Age, and Sex in the US. *JAMA Netw Open*. Published February 27, 2025. doi:10.1001/jamanetworkopen.2024.62069

Data
Data available: Yes
Data types: Deidentified participant data
How to access data: https://vizhub.healthdata.org/subnational/usa
When available: With publication

Supporting Documents
Document types: None

Additional Information
Who can access the data: Data will be publicly available
Types of analyses: Any purpose
Mechanisms of data availability: With or without investigator support
